# N6-methyladenosine RNA modification regulates the transcription of SLC7A11 through KDM6B and GATA3 to modulate ferroptosis

**DOI:** 10.1186/s12929-024-01100-y

**Published:** 2025-01-13

**Authors:** Haisheng Zhang, Cheng Yi, Jianing Li, Yunqing Lu, Haoran Wang, Lijun Tao, Jiawang Zhou, Yonghuang Tan, Jiexin Li, Zhuojia Chen, Gholamreza Asadikaram, Jie Cao, Jianxin Peng, Wanglin Li, Junming He, Hongsheng Wang

**Affiliations:** 1 Guangdong Provincial Key Laboratory of New Drug Design and Evaluation, State Key Laboratory of Anti-Infective Drug Discovery and Development, School of Pharmaceutical Sciences, Sun Yat-Sen University, Guangzhou, 510006 China; 2 State Key Laboratory of Oncology in South China, Collaborative Innovation Center for Cancer Medicine, Sun Yat-Sen University Cancer Center, Guangzhou, 510060 China; 3 Endocrinology and Metabolism Research Center, Institute of Basic and Clinical Physiology Sciences, Kerman University of Medical Sciences, Medical University Campus, Kerman, Iran; 4 Department of General Surgery, Guangzhou Digestive Disease Center, Guangzhou First People’s Hospital, The Second Affiliated Hospital of South China University of Technology, Guangzhou, 510180 China; 5 Department of Hepatobiliary Surgery, Guangdong Province Traditional Chinese Medical Hospital, Guangzhou, 510120 China; 6 Huadu District People’s Hospital of Guangzhou, Guangzhou, 510800 China

**Keywords:** METTL3, SLC7A11, KDM6B, GATA3, Ferroptosis

## Abstract

**Background:**

Recent studies indicate that N6-methyladenosine (m^6^A) RNA modification may regulate ferroptosis in cancer cells, while its molecular mechanisms require further investigation.

**Methods:**

Liquid Chromatography-Tandem Mass Spectrometry (HPLC/MS/MS) was used to detect changes in m^6^A levels in cells. Transmission electron microscopy and flow cytometry were used to detect mitochondrial reactive oxygen species (ROS). RNA sequencing (RNA-seq) was employed to analyze the factors regulating ferroptosis. Chromatin immunoprecipitation (ChIP) was used to assess the binding of regulatory factors to the SLC7A11 promoter, and a Dual-Luciferase reporter assay measured promoter activity of SLC7A11. The dm^6^ACRISPR system was utilized for the demethylation of specific transcripts. The Cancer Genome Atlas Program (TCGA) database and immunohistochemistry validated the role of the METTL3/SLC7A11 axis in cancer progression.

**Results:**

The m^6^A methyltransferase METTL3 was upregulated during cancer cell ferroptosis and facilitated erastin-induced ferroptosis by enhancing mitochondrial ROS. Mechanistic studies showed that METTL3 negatively regulated the transcription and promoter activity of SLC7A11. Specifically, METTL3 induced H3K27 trimethylation of the SLC7A11 promoter by suppressing the mRNA stability of H3K27 demethylases KDM6B. Furthermore, METTL3 suppressed the expression of GATA3, which regulated SLC7A11 transcription by binding to the putative site at − 597 to − 590 of the SLC7A11 promoter. METTL3 decreased the precursor mRNA stability of GATA3 through m^6^A/YTHDF2-dependent recruitment of the 3′-5′ exoribonuclease Dis3L2. Targeted demethylation of KDM6B and GATA3 m^6^A using the dm^6^ACRISPR system significantly increased the expression of SLC7A11. Moreover, the transcription factor YY1 was responsible for erastin-induced upregulation of METTL3 by binding to its promoter-proximal site. In vivo and clinical data supported the positive roles of the METTL3/SLC7A11 axis in tumor growth and progression.

**Conclusions:**

METTL3 regulated the transcription of SLC7A11 through GATA3 and KDM6B to modulate ferroptosis in an m^6^A-dependent manner. This study provides a novel potential strategy and experimental support for the future treatment of cancer.

**Supplementary Information:**

The online version contains supplementary material available at 10.1186/s12929-024-01100-y.

## Background

N6-methyladenosine (m^6^A) stands as the most prevalent modification found within eukaryotic mRNAs [1]. This dynamic modification is mediated by the m^6^A methyltransferase complex (MTC), which includes methyltransferase-like 3 (METTL3), methyltransferase like 14 (METTL14), and Wilms’ tumor 1-associating protein (WTAP). Conversely, the removal of m^6^A is facilitated by RNA demethylases, including fat mass and obesity-associated protein (FTO) and Alkb homolog 5 (ALKBH5) [2, 3]. m^6^A is recognized by various reader proteins, predominantly comprising YT521-B homology domain-containing proteins (YTHDFs), insulin-like growth factor 2 mRNA-binding proteins (IGF2RPs), and RNA-binding proteins [4–6]. m^6^A regulates pre-mRNA splicing and nuclear export, affects mRNA stability and translation, and influences various biological and physiological functions, including cancer development [2, 7, 8].

Ferroptosis is a newly identified form of programmed cell death, distinct from apoptosis and other forms of cell death [9]. It is an iron-dependent cell death characterized by the accumulation of lipid peroxidation and impairment of antioxidant systems, resulting in shrunken mitochondria and reduced numbers of mitochondrial cristae [10]. The classical small molecule erastin, known to induce ferroptosis, inhibits the amino acid transporter solute carrier family 7 member 11 (SLC7A11), leading to a reduction in glutathione. Additionally, erastin can inactivate the phospholipid peroxidase glutathione peroxidase 4 (GPX4), resulting in the accumulation of lipid oxidation products, similarly to RSL3 [11]. Downregulation or inhibition of SLC7A11 and GPX4 can lead to a loss of intracellular cystine levels and subsequently activate ferroptosis [12, 13].

Recent studies have indicated that epigenetic factors, including RNA methylation, are important regulators of cancer cell ferroptosis [13]. For example, m^6^A-regulated FGFR4 attenuates ferroptotic cell death in recalcitrant HER2-positive breast cancer [14]. IGF2BP3 is an essential m^6^A target for suppressing ferroptosis in lung adenocarcinoma cells [15], while METTL16 epigenetically enhances GPX4 expression via m^6^A modification to promote breast cancer progression by inhibiting ferroptosis [16]. Considering the increasing number of studies revealing the essential roles of m^6^A in cancer progression, further investigations are needed to understand the potential roles and related mechanisms of m^6^A in cancer-related ferroptosis.

The roles of m^6^A and METTL3 in ferroptosis are not consistent in cancer cells. METTL3 has been reported as a ferroptosis inhibitor by upregulating of SLC7A11 [17–19]. However, METTL3 also mediates erastin-induced ferroptosis in renal cancer stem cells by promoting m^6^A modification of ALOX12/P53 mRNA [20]. Regarding the regulation of SLC7A11, METTL3 promotes lung adenocarcinoma (LUAD) tumor growth and inhibits ferroptosis by stabilizing the m^6^A modification of SLC7A11 [17]. However, METTL3 destabilizes m^6^A-methylated SLC7A11 mRNA to impair cystine uptake in LUAD [21]. m^6^A peaks are abundant in the 3′UTR of SLC7A11 mRNA just near the stop codons, and there is no m^6^A peak in 5′UTR [21]. Nevertheless, it has also been reported that m^6^A methylation at 5′UTR of SLC7A11 mRNA in HCC [22]. The regulation of SLC7A11 expression by m^6^A seems to be diverse.

Preliminary findings suggest that erastin induces an increase in cellular m^6^A levels by upregulating METTL3. Considering the significance of m^6^A and ferroptosis in cancer cells, we aim to explore the underlying mechanisms. Our data indicate that METTL3 controls SLC7A11 transcription through GATA binding protein 3 (GATA3) and H3K27 lysine demethylase 6B (KDM6B), highlighting a potential approach for cancer therapy by disrupting m^6^A-regulated ferroptosis in cancer cells.

## Materials and methods

### Cell culture and reagents

Human lung cancer cell A549, cervical cancer cell HeLa and SiHa, breast cancer MDA-MB-231 were commercially obtained from ATCC and maintained by our laboratory. We used DMEM for HeLa and SiHa cells, RPMI 1640 for MDA-MB-231 and A549 cells. Cells in the culture medium were supplemented with 10% FBS (Procell, China) and 100 U/mL penicillin/streptomycin (Beyotime, China) under an atmosphere of 5% CO_2_ at 37 °C. Erastin (HY-15763), ferrostatin-1 (HY-100579) and GSK-J4 (HY-15648B) were purchased from MedChemExpress (China). Z-VAD-FMK (GC12861), Necrosulfonamide (NSA, GC10150) and Acetylcysteine (NAC, GC11786) were purchased from Glpbio (America).

### Plasmid, siRNA, shRNA and generation of stable cell lines

Stable knockdown of METTL3 cell lines was generated by CRISPR-cas9 editing system. sgRNA sequence of 5′-TCT GAA CCA ACA GTC CAC TA-3′ was inserted into pSpCas9(BB)−2A-Puro (PX459) V2.0. Positive clones were screened using 1 μg/mL puromycin after transfection of sgRNA of METLL3 into cells. Based on the sequencing results, monoclonal clones with effective mutations were selected. Stable knockdown cell lines were detected using western blot, and were used for subsequent experiments after expansion. To generate sh-METTL3 stable cell lines, a target sequence of 5′-GCT GCA CTT CAG ACG AAT T-3′ was inserted into pCLenti-U6-shRNA (METTL3)-CMV-Puro-WPRE which was completed in Obio (China). Then cells cultured on complete medium and maintained with 1 μg/mL puromycin. METTL3 and METTL3 mutant DA(D395A) plasmids were generated in our previous study. Briefly, the CDS of METTL3 and SLC7A11 were cloned into pPB to generate overexpression plasmid. Targets of interfering sequences are listed in Table S4.

### Cell viability assay

Cell Counting Kit-8(NCM Biotech, C6005) was used to evaluate cell viability according to the manufacturer’s protocol. Briefly, cells were seeded on 96-well plates at 1 × 10^4^ cells per well in 10% FBS-supplemented medium before treatment with drugs. After treatment at indicated conditions, 10 μL of CCK-8 solution was added to each well. The plates were incubated at 37 °C for another 2 h, and the absorbance was measured at 450 nm using a microplate reader.

### Western-blot analysis

Cells were washed three times with ice-cold phosphate buffer solution (PBS) and then lysed in lysis buffer (Beyotime, P0013) for 10 min. Lysates were cleared by centrifugation. Equal amounts of protein samples were loaded per well and separated on sodium dodecyl sulfate–polyacrylamide gel electrophoresis (SDS-PAGE), and then electrophoretically transferred onto polyvinylidene fluoride (PVDF) membranes. Following blocking with 5% non-fat milk at room temperature for 2 h, the membranes were incubated with primary antibodies at 4 °C overnight and then incubated with horseradish peroxidase (HRP)-conjugated secondary antibodies for 2 h at room temperature. Specific immune complexes were detected using Femto-Sensitive ECL Solution (MIKX, MK-01000). The relative grey level of proteins was calculated using Image J software. The antibodies used in this study for western blot analysis are listed in Table S5.

### Quantitative RT-PCR

Total RNA was extracted from cells using TRIzol Reagent (Agbio, AG21102) and reverse transcribed by Evo M-MLV (Agbio, AG11706). Quantitative RT-PCR was performed with SYBR Green (Agbio, AG11701) using CFX Manager 3.1(Bio-Rad) as recommended by the manufacturer’s protocol. The sequences of primers are listed in Table S6.

### Dot blot

After incubating at 95 °C for 5 min, poly (A) + RNAs were added to nylon N + membrane (Biosharp, BS-NY-45). After UV crosslinking for 30 min, the membrane was stained with 0.02% methylene blue for 2 h. After taking photos and washing the methylene off, the membrane was blocked in 5% milk. Then the membrane was incubated with m^6^A antibody overnight at 4 °C. After incubating with the m^6^A antibody at room temperature for 2 h, the membrane was treated with ECL for capture.

### Transmission electron microscopy

HeLa WT or HeLa METTL3 KD cells were plated at 1 × 10^6^ cells/dish in 100 mm cell culture dishes. Then the cells were treated with DMSO or erastin (2 μM) for 24 h. Cells were fixed with 2.5% glutaraldehyde (Servicebio, G1102). A transmission electron microscopy was used to acquire the images. This assay was completed in Landmbio (China).

### Lipid peroxidation assay

Cells were seeded in a 6-well culture plate at 2 × 10^5^ cells per well. Cells were treated with indicated conditions, and then the culture medium was replaced with 500 μL medium containing 5 μM of BODIPY-C11(581/591) (#D3861, Invitrogen). The plate was returned to the cell culture incubator for another 30 min. After washing three times with PBS, cells were digested with trypsin and resuspended in 500 μL of fresh PBS. Cells were filtered through a cell strainer and analyzed using a flow cytometer. Data analysis was performed using FlowJo software.

### Protein and mRNA stability

To measure protein stability, cells were treated with cycloheximide (final concentration 20 μg/mL, Cayman, Catalog #14,126) during indicated times. The expression of the proteins was measured through western blot analysis.

To measure RNA stability in cells, actinomycin D (Act-D, Catalog #GC16866, Glpbio, USA) at 5 μg/mL was added to the cells. After incubation at the indicated times, cells were collected, and RNA was extracted for real-time PCR. Relative expression of precursor or mature mRNA was calculated and GAPDH was used for normalization.

### m^6^A-RIP-qPCR

Total RNA was extracted using TRIzol and fragmented by RNA fragmentation reagents (Thermo, AM8740) or not. After saving 50 ng of the total RNA as input, the remaining RNAs (200 μg) were used for m^6^A-immunoprecipitation with an m^6^A antibody (Synaptic Systems) in 500 μL of IP buffer (150 mM NaCl, 0.1% NP-40, 10 mM Tris, pH 7.4, 100 U RNase inhibitor) to obtain the m^6^A pull down portion (m^6^A IP portion). m^6^A RNAs were immunoprecipitated with Dynabeads® Protein A (ThermoFisher Scientific) and eluted twice with an elution buffer (5 mM Tris–HCL pH 7.5, 1 mM EDTA pH 8.0, 0.05% SDS, 20 mg/mL Proteinase K). m^6^A IP RNAs were recovered by ethanol precipitation, and RNA concentration was measured with Qubit® RNA HS Assay Kit (ThermoFisher Scientific). Then 2 ng of both total RNA and m^6^A IP RNA were used as templates in qRT-PCR, as described above.

### Immunohistochemistry (IHC)

Tumor tissues were fixed in formalin and embedded in paraffin. For immunohistochemical staining, sections were deparaffinized and hydrated. Endogenous peroxidase activity was blocked with 3% H_2_O_2_ in water for 20 min. Antigen retrieval was performed with 10 mM citrate buffer (pH 6.0) for 30 min. Slides were incubated with goat serum (BOSTER) for 60 min to block nonspecific binding. Then, slides were incubated with primary antibodies overnight at 4 °C, washed with PBS twice, and then incubated with goat anti-rabbit HRP-conjugated secondary antibodies for 2 h at room temperature. Finally, slides were incubated with 3,3’-diami-nobenzidine and counterstained with hematoxylin.

### Chromatin Immunoprecipitation (ChIP)

ChIP analysis was performed as previously reported [23]. In brief, cross-linking was completed after cell culture, followed by nuclei preparation and chromatin digestion. Samples immunoprecipitated with a normal IgG and a specific antibody were purified and subjected to qPCR. The sequences of the primers are listed in Table S6.

### ChIP-Atlas enrichment analysis

The ChIP-Atlas [24] database parameters were set as follows: “*H. sapiens*(hg19)”; “ChIP: TFs and others” for Experiment Type; “Breast” for Cell Type Class; “100” for Threshold for Significance; “SLC7A11” for Enter Dataset; “ − 1000 bp ≤ transcription start site (TSS) ≤ + 1000 bp” for the distance range from TSS. *P*-values were calculated using the two-tailed Fisher's exact probability test.

### Xenograph mouse model

About 5 × 10^6^ of MDA-MB-231 sh-NC or sh-METTL3 cells were injected subcutaneously into each 4-week-old immunodeficient BALB/c nude mice. Tumor formation was monitored, and tumor volume was calculated by the formula: tumor volume = 1/2(length × width^2^). When the tumors reached a volume of approximately 150 mm^3^, mice were randomly allocated into different groups (final five mice in each group) and treated with erastin (30 mg/kg, every day) or vehicle (5% dimethylsulfoxide (DMSO) in corn oil) for 16 days. Tumors were isolated from mice and weighed at the end of the experiment. The animal experiment was approved by the Institutional Animal Care and Use Committee (IACUC), Sun Yat-Sen University (No. SYSU-IACUC-2022–001263).

### Iron and MDA assay

The iron accumulation was detected using the iron assay kit (Solarbio, BC4355/BC5315). ① Preparation of reagents and standards prior to use; ② Approximately 0.1 g of tissue was weighed and homogenized in 1 mL of extraction buffer on ice. The homogenate was centrifuged at 4000*g* at 4 °C for 10 min, and the supernatant was collected; ③ Addition of reagents followed by thorough mixing and centrifugation at room temperature at 10,000 rpm for 10 min. The upper aqueous phase (200 μL) was carefully transferred to a microplate or 96-well plate, and absorbance was immediately measured at 520 nm; ④Reduction of Fe^3+^ to Fe^2+^ using sodium bisulfite, followed by complexation with 2,2′-bipyridine resulting in an absorption peak at 520 nm. Absorbance at this wavelength was measured, and iron content was calculated based on tissue protein concentration.

The relative MDA concentration was assessed using a Microscale Malondialdehyde (MDA) assay kit (A003-2, Nanjing Jiancheng Bioengineering Institute, Nanjing, China) according to the manufacturer’s instructions. Briefly, MDA reacted with thiobarbituric acid (TBA) at 90–100 °C and acidic conditions to generate a pink MDA-TBA conjugate, which was measured at 532 nm.

### Database (DB) search

We used the Kaplan–Meier database (http://kmplot.com/analysis/) to test the overall survival (OS) of METTL3/SLC7A11 axis. The difference between survival curves was determined by the log-rank test, including its *p*-value. Expression of METTL3 protein in BC tissues and other proteins such as SLC7A11 were extracted from TCGA database.

### Statistical analyses

Data were reported as mean ± SD from at least three independent experiments. For statistical analysis, a two-tailed unpaired Student's t-test was conducted between two groups, and one-way or two-way ANOVA followed by the Bonferroni test for multiple comparisons was performed. All statistical tests were two-sided. Data analysis was carried out using SPSS 16.0 for Windows. A *p*-value of < 0.05 was considered statistically significant. *p < 0.05, **p < 0.01, ***p < 0.001; ns, no significant.

## Results

### METTL3 facilitated erastin-induced ferroptosis and enhanced mitochondrial ROS

**Fig. 1:**
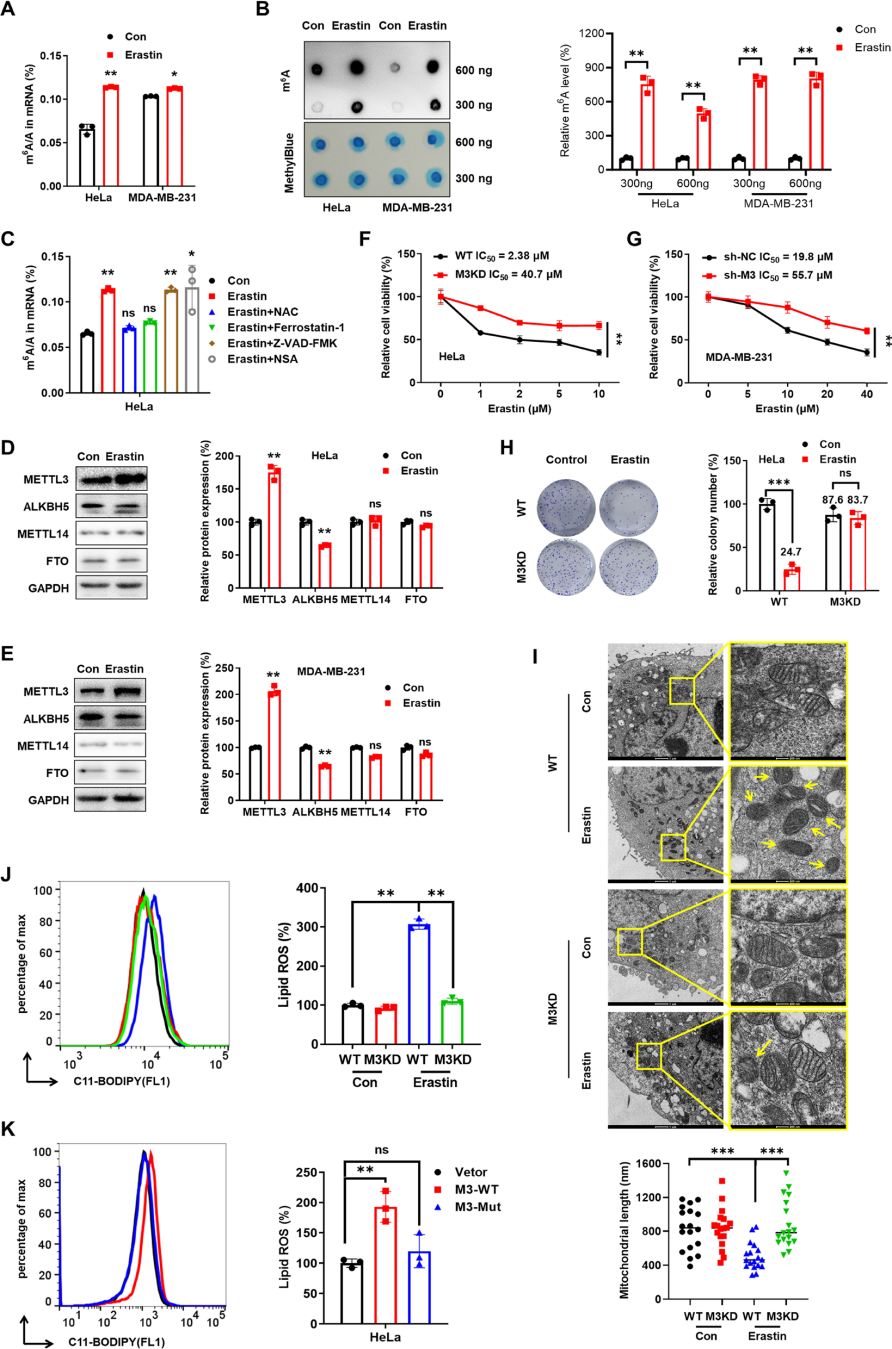
METTL3 facilitated erastin-induced ferroptosis and enhanced mitochondrial ROS.** A** Cells were treated with or without 2 μM erastin for 24 h, and the m^6^A/A ratios of mRNA were checked by HPLC/MS/MS. **B** Dot blot (*left*) and quantitative analysis (*right*) were used to detect the total mRNA m^6^A. **C** HeLa cells were treated with erastin (2 μM), ferrostatin-1 (1 μM), acetylcysteine (NAC, 2 mM), Z-VAD-FMK (10 μM) and necrosulfonamide (NSA, 5 μM) for 24 h, and the m^6^A/A ratios of mRNA were checked by HPLC/MS/MS. **D**, **E** HeLa (**D**) and MDA-MB-231 (**E**) cells were treated with 2 μM erastin for 24 h, and the protein expression was assessed by western blot (*left*) and quantitative analysis (*right*). **F**, **G** HeLa (**F**) and MDA-MB-231 (**G**) cells were treated with increasing concentrations erastin for 48 h, and the relative cell viability was detected using Cell Counting Kit-8 kit. **H** HeLa cells were treated with or without erastin (0.1 μM) for 14 days, and the colonization capability was evaluated (*left*) and analyzed (*right*). **I** Representative transmission electron microscopy images of WT or METTL3 KD HeLa cells treated with erastin (2 μM, 24 h) (*up*). Yellow arrow represent shrunken mitochondria. The length of mitochondria was measured by image J (*down*). **J** WT and METTL3 KD HeLa cells were treated with or without erastin (2 μM) for 24 h, and lipid ROS production was assayed by flow cytometry using C11-BODIPY. **K** HeLa cells were transfected with vector control, METTL3 WT plasmid, or METTL3 DA mutant plasmid for 24 h, and lipid ROS production was assayed by flow cytometry using C11-BODIPY. Data are presented as mean ± SD from three independent experiments. *p < 0.05, **p < 0.01, ***p < 0.001, ns, no significant, by Student’s* t* test between two groups and by one-way ANOVA followed by Bonferroni test for multiple comparison

To determine if m^6^A is involved in ferroptosis, we treated cancer cells with erastin, which inhibits cystine uptake [9]. LC/MS/MS analysis revealed that m^6^A levels in mRNAs from erastin-treated HeLa and MDA-MB-231 cells were significantly higher than in control cells (Fig. 1 A). Similarly, the m^6^A levels of mRNAs were increased by sorafenib and RSL3 (Figure S1 A), two additional ferroptosis-inducing agents, as well as by erastin, which upregulated m^6^A levels in SiHa and A549 cells (Figure S1 B). Dot-blot assays confirmed these findings (Fig. 1 B). In addition, the increased m^6^A levels of mRNA were blocked by ferrostatin-1 (a potent ferroptosis inhibitor) and NAC (the precursor of intracellular antioxidant GSH), but not by Z-VAD-FMK (a potent apoptosis inhibitor) and necrosulfonamide (NSA, a potent necroptosis inhibitor) (Fig. 1C) [25]. This indicates that the m^6^A modification of mRNA in cancer cells increases during erastin-induced ferroptosis.

To investigate the regulators responsible for erastin-induced m^6^A upregulation, we checked the expression of methyltransferases METTL3/METTL14, as well as demethylases ALKBH5/FTO. Western blot analysis showed that erastin significantly increased METTL3 expression and decreased ALKBH5 expression, with no effect on METTL14 or FTO in HeLa (Fig. 1D) and MDA-MB-231 cells (Fig. 1E). In addition, erastin increased METTL3 expression and decreased ALKBH5 expression in a concentration-dependent manner in both cell lines (Figure S1C). These results suggest that erastin upregulates METTL3 and downregulates ALKBH5 in cancer cells.

To determine if METTL3 and/or ALKBH5-regulated m^6^A variations are involved in erastin-induced ferroptosis, we examined potential effects of erastin on stable knockdown METTL3 in HeLa and MDA-MB-231 cells. The knockdown of METTL3 suppressed the proliferation of both HeLa and MDA-MB-231 cells (Figure S1D), but significantly decreased the sensitivity to erastin-induced ferroptosis (Fig. 1F, G). Moreover, the knockdown of METTL3 significantly attenuated erastin-suppressed cell colonization of both HeLa (Fig. 1H) and MDA-MB-231 cells (Figure S1E). Overexpression of METTL3, but not the catalytically inactive METTL3 mutant DA (D395A) [26], increased the sensitivity to erastin-induced death (Figure S1F). Functionally, METTL3 overexpression promoted erastin-induced cell death, which was prevented by ferrostatin-1 and NAC, while Z-VAD-FMK and NSA did not suppress the erastin-triggered increase in cell death in METTL3-overexpressing HeLa cells (Figure S1G, H).

Overexpression of ALKBH5 had no effect on erastin-induced death (Figure S1I–K) and did not significantly impact erastin-suppressed cell colonization (Figure S1L). This suggests that the alteration of ALKBH5 expression during erastin-induced ferroptosis of cancer cells might be a concomitant phenomenon. Recent studies have reported that METTL4, another methyltransferase, is involved in the upregulation of m^6^A modification during ferroptosis [27, 28]. Therefore, we knocked down METTL4 using sh-RNA in HeLa and MDA-MB-231 cells (Figure S1M). This resulted in reduced erastin-induced redox-active iron accumulation (Figure S1N), while erastin did not affect METTL4 expression (Figure S1O). Collectively, these data suggest that METTL3, rather than ALKBH5 or METTL4, is responsible for m^6^A-triggered erastin-induced ferroptosis in cancer cells.

Mitochondrial ROS are key factors in inducing both apoptosis and ferroptosis [29]. To verify their involvement in METTL3-facilitated ferroptosis, we analyzed mitochondrial morphology using transmission electron microscopy. Our data showed that erastin-treated cells exhibited shrunken mitochondria with increased membrane density, while these effects were dramatically suppressed by the knockdown of METTL3 (Fig. 1I). Additionally, METTL3 knockdown significantly suppressed erastin-induced accumulation of lipid ROS, a biomarker of ferroptosis, which was determined by flow cytometry using the fluorescent probe C11-BODIPY (Figs. 1J, S1P). Overexpression of METTL3, but not the inactive mutant, notably increased erastin-triggered lipid peroxidation in HeLa cells (Fig. 1K). Erastin reduced GSH levels, while METTL3 knockdown increased them, and overexpression decreased GSH in HeLa and MDA-MB-231 cells (Figure S1Q–T). Similarly, METTL3 KD in HeLa cells increased intracellular cysteine levels (Figure S1U). METTL3 KD significantly attenuated erastin-induced iron and MDA accumulation (Figure S1V, W). Notably, treatment with Mito-TEMPO partially reversed the iron and MDA accumulation induced by erastin and mitigated the effects of METTL3 KD (Figure S1V, W). Together, our data suggest that METTL3 facilitated erastin-induced ferroptosis via enhancing mitochondrial ROS.

### METTL3 triggered ferroptosis via suppressing SLC7A11 transcription

**Fig. 2:**
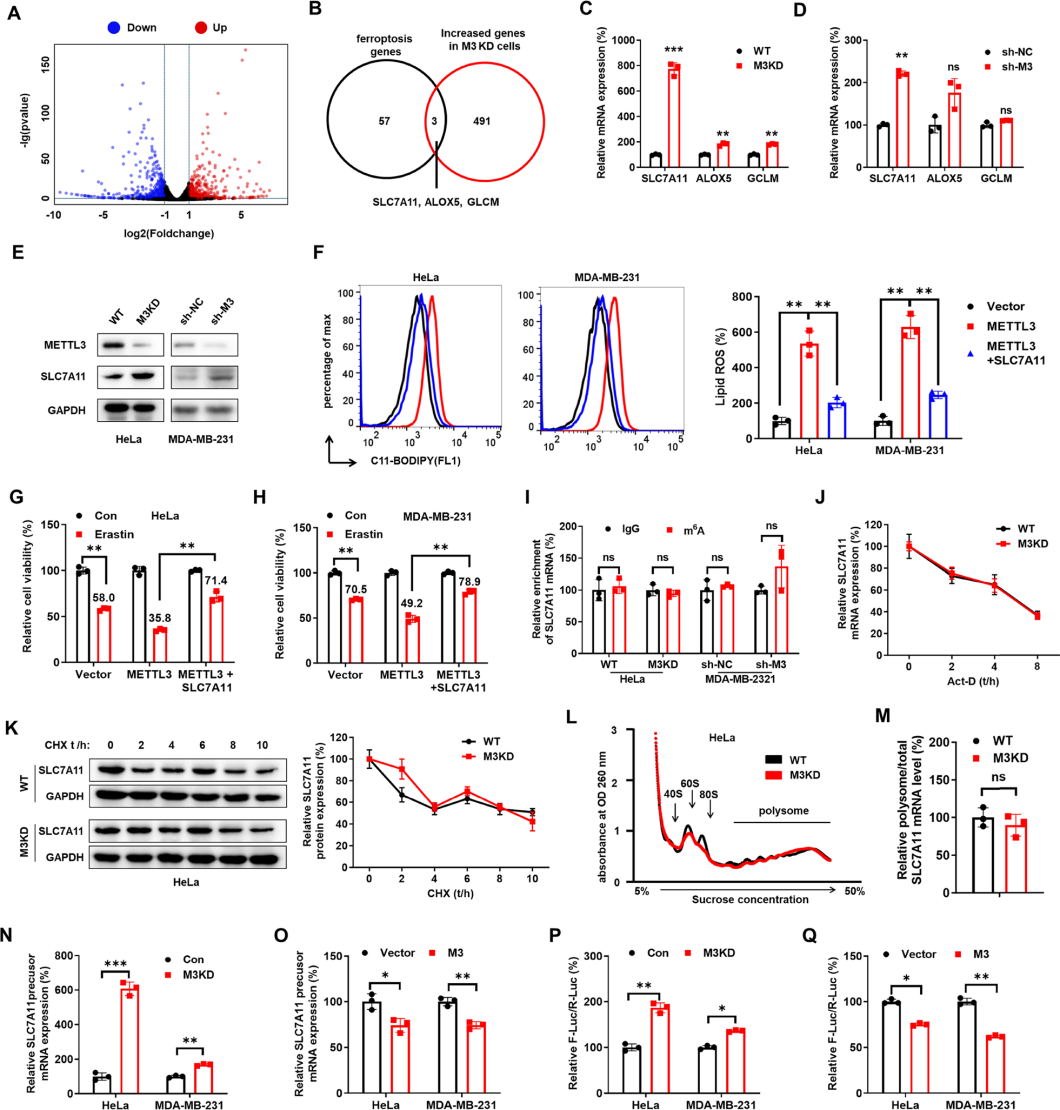
METTL3 triggered ferroptosis via suppressing SLC7A11 transcription. **A** The volcano plot showed the mutated genes between WT and METTL3 KD cells. **B** The overlap between the genes involved in ferroptosis and the mutated genes in METTL3 KD cells. **C**, **D** The mRNA expression of SLC7A11, ALOX5 and GLCM in HeLa (**C**) and MDA-MB-231 (**D**) was checked by qRT-PCR. **E** The protein expression of SLC7A11 in HeLa and MDA-MB-231 cells was checked by western blot analysis. **F** HeLa and MDA-MB-231 cells were transfected with vector control, METTL3 plasmid, and SLC7A11 plasmid for 24 h, and lipid ROS production was assayed by flow cytometry using C11-BODIPY. **G**, **H** HeLa (**G**) and MDA-MB-231 (**H**) HeLa cells were transfected with vector control, METTL3 plasmid, and SLC7A11 plasmid for 24 h, and the relative cell viability was detected using Cell Counting Kit-8 kit after treatment with erastin for 48 h. **I** m^6^A RIP-qPCR analysis of SLC7A11 mRNA in HeLa and MDA-MB-231 cells. **J** After treatment with Act-D for the indicated times, the mature mRNA levels of SLC7A11 were checked in WT and METTL3 KD HeLa cells by qRT-PCR. **K** WT or METTL3 KD HeLa cells were treated with 10 μg/ml CHX for the indicated time periods, and then the protein expression of SLC7A11 was detected by western blot (*left*) and quantitatively analyzed (*right*). **L** Polysome profiling of WT or METTL3 KD HeLa cells was analyzed. **M** Analysis of SLC7A11 mRNA in polysome/total for the METTL3 KD cells compared to control cells. **N**, **O** The SLC7A11 precursor mRNA expression HeLa and MDA-MB-231cells was checked by qRT-PCR. **P**, **Q** The luciferase activity of the SLC7A11 promoter in the F-Luc/R-Luc system was measured using the Dual-Lumi™ II Luciferase Assay Kit. Data are presented as mean ± SD from three independent experiments. *p < 0.05, **p < 0.01, ***p < 0.001, ns, no significant, by Student’s* t* test between two groups and by one-way ANOVA followed by Bonferroni test for multiple comparison

To characterize potential targets involved in METTL3-regulated ferroptosis, we performed mRNA-seq in wild-type and METTL3 KD HeLa cells [30]. The expression levels of 949 genes were found to be significantly altered, with the upregulation of 494 genes and downregulation of 455 genes in METTL3 KD HeLa cells (Fig. 2A). Gene Set Enrichment Analysis (GSEA) revealed that the gene expression profiles of adipogenesis (Figure S2A) and the metabolisms of butanoate, glycerophospholipid, retinol, ascorbate and aldarate metabolism (Figure S2B–E) were significantly increased in METTL3 KD HeLa cells as compared with those in HeLa cells. The transcriptome study revealed that METTL3 KD HeLa cells can reprogram lipid-related metabolism and ROS generation pathways.

We characterized potential targets involved in m^6^A-regulated ferroptosis of cancer cells. The knockdown of METTL3 resulted in erastin-resistant cancer cells, prompting us to focus on upregulated genes in METTL3 knockdown HeLa cells. Among 60 ferroptosis-related genes (Table S1), we identified three candidates SLC7A11, ALOX5 and GCLM, that were significantly increased in METTL3 KD HeLa cells (p < 0.05) (Fig. 2B). qPCR confirmed the upregulation of SLC7A11, ALOX5 and GCLM in METTL3 KD HeLa cells (Fig. 2C), while only SLC7A11 was upregulated in MDA-MB-231 sh-METTL3 cells (Fig. 2D). SLC7A11, a key component of the cystine/glutamate antiporter that suppresses ferroptosis [31], was increased in METTL3 KD HeLa and sh-METTL3 MDA-MB-231 cells (Fig. 2E). The knockdown of METTL3 also increased SLC7A11 in SiHa and A549 cells (Figure S2F). Overexpression of METTL3, but not the DA (D395A) mutant, suppressed SLC7A11 expression (Figure S2G). These findings suggest that METTL3 negatively regulates SLC7A11 in cancer cells. Despite SLC7A11’s established role in ferroptosis, we investigated its involvement in m^6^A-regulated ferroptosis. Co-transfection of METTL3 and SLC7A11 constructs showed that SLC7A11 overexpression attenuated METTL3-induced fluorescence intensity of C11-BODIPY (Fig. 2F) and reduced sensitivity to erastin in METTL3-overexpressing cancer cells (Fig. 2G, H). Thus, METTL3 primarily promotes ferroptosis by repressing SLC7A11 expression.

We then investigated mechanisms responsible for m^6^A regulated expression of SLC7A11. First, we checked whether SLC7A11 mRNA was directly modified by m^6^A. There was no significant enrichment of m^6^A in SLC7A11 mRNA in HeLa cells (Figure S2I). Furthermore, SRAMP [32] prediction result suggested one confidence m^6^A site on SLC7A11 mRNA (Figure S2J) [21]. However, m^6^A-RIP-qPCR confirmed that no significant m^6^A modification enriching SLC7A11 mRNA in HeLa and MDA-MB-231 cells (Fig. 2I). This suggested that METTL3-regulated expression of SLC7A11 might not directly methylate its mRNA.

We then investigated the potential mechanism responsible for METTL3-regulated mRNA expression of SLC7A11. First, there is no significant difference in the stability of mature-mRNA in both METTL3 KD HeLa (Fig. 2J) and sh-METTL3 MDA-MB-231 (Figure S2 K) cells. It indicated that METTL3-regulated expression of SLC7A11 might not be due to mRNA stability. Further, half-lives of SLC7A11 protein were similar between wild-type and METTL3 KD HeLa cells (Fig. 2K) or between sh-NC and sh-METTL3 MDA-MB-231 cells (Figure S2 L), suggesting that m^6^A-regulated SLC7A11 expression was not related to protein stability. Next, we isolated the non-translating fraction (< 40S), translation initiation fraction (including 40S and 60S ribosomes, 80S monosomes, < 80S) and translation active polysomes (> 80S) (Fig. 2L). qPCR showed SLC7A11 mRNA in translation active polysomes (> 80S) of METTL3 KD HeLa cells was increased as compared with that in wild-type cells (Figure S2M), but the up-regulation ratio was the same as that of the mRNA (Fig. 2M). It indicated that METTL3-regulated expression of SLC7A11 might not be due to translational or post-translational regulation.

We then investigated whether METTL3-regulated expression of SLC7A11 was due to transcription. qRT-PCR showed that the precursor mRNA of SLC7A11 was increased in METTL3 knockdown cells (Fig. 2N), and overexpression of METTL3 decreased the precursor-mRNA of SLC7A11(Fig. 2O). To further confirm that the transcription activity of SLC7A11 was regulated by METTL3, we sub-cloned its promoter to the pGL3-Basis luciferase reporter [33]. Dual luciferase assay showed that the promoter activities of SLC7A11 in METTL3 KD HeLa or sh-METTL3 MDA-MB-231 cells were significantly greater than that in their control cells (Fig. 2P). Furthermore, overexpression of METTL3 significantly decreased the promoter activity of SLC7A11 in both HeLa and MDA-MB-231 cells (Fig. 2Q). All these data confirmed that METTL3 negatively regulates the transcription of SLC7A11.

### METTL3 induced the H3K27 trimethylation of SLC7A11 promoter via suppression of KDM6B

**Fig. 3:**
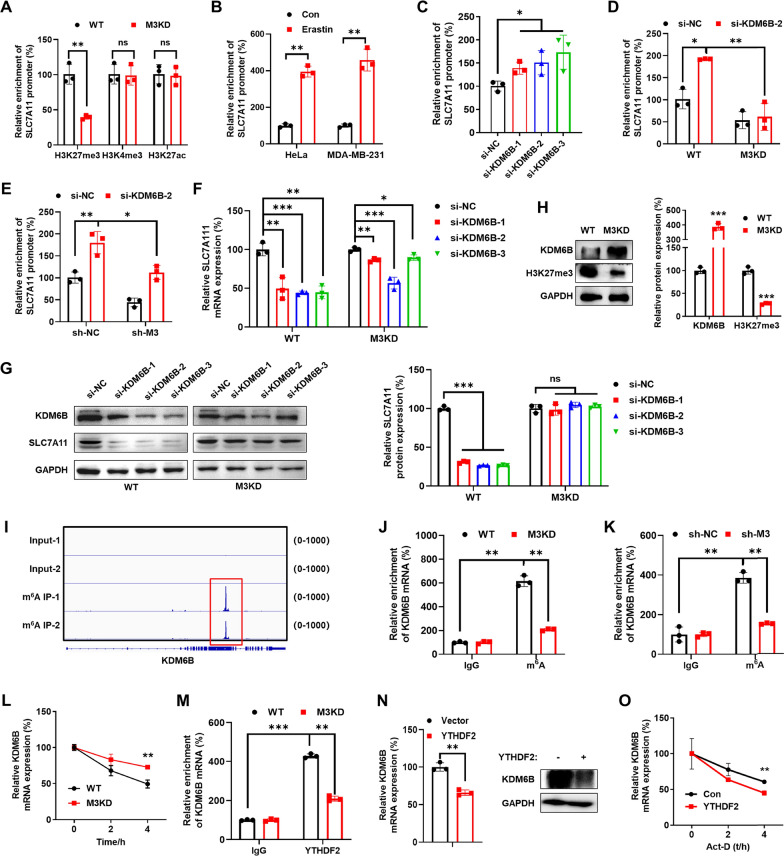
METTL3 induced the H3K27 trimethylation of SLC7A11 promoter via suppression of KDM6B. **A** ChIP-qPCR assay was performed to examine H3K27me3, H3K4me3 and H3K27ac binding to the SLC7A11 promoter in HeLa cells. **B** After treating HeLa (*left*) and MDA-MB-231 cells (*right*) with 2 μM erastin, ChIP-qPCR analysis was performed to examine the binding of H3K27me3 at the promoter region of SLC7A11. **C** ChIP-qPCR analysis was performed to examine the binding of H3K27me3 at the promoter region of SLC7A11 after transfecting with siRNAs of KDM6B for 24 h in HeLa WT cells. **D** ChIP-qPCR analysis was performed to examine the binding of H3K27me3 at the promoter region of SLC7A11 after transfecting with siRNAs of KDM6B for 24 h in HeLa WT and METTL3 KD cells. **E** ChIP-qPCR analysis was performed to examine the binding of H3K27me3 at the promoter region of SLC7A11 after transfecting with siRNAs of KDM6B for 24 h in MDA-MB-231 sh-NC and sh-METTL3 cells. **F** SLC7A11 mRNA was detected after transfecting with siRNAs of KDM6B for 24 h in HeLa cells by qRT-PCR. **G** SLC7A11 and KDM6B proteins were detected after transfecting with siRNAs of KDM6B for 48 h in HeLa cells by western blot (*left*) and quantitatively analyzed (*right*). **H** H3K27me3 and KDM6B proteins were detected by western blot (*left*) and quantitatively analyzed (*right*) in HeLa WT and METTL3 KD cells. **I** One m^6^A peak was enriched in KDM6B from m^6^A RIP-seq data. **J** m^6^A RIP-qPCR analysis of KDM6B mRNA in HeLa WT or METTL3 KD cells. **K** m^6^A RIP-qPCR analysis of KDM6B mRNA in MDA-MB-231 sh-NC or sh-METTL3 cells. **L** After treatment with Act-D for the indicated times, the mature mRNA levels of KDM6B were checked in WT and METTL3 KD HeLa cells. **M** YTHDF2 RIP-qPCR analysis of KDM6B mRNA in WT or METTL3 KD HeLa cells. **N** KDM6B protein was detected after transfecting with vector or YTHDF2 plasmid for 48 h in HeLa cells by western blot analysis (*right*), and mRNA by qRT-PCR (*left*). **O** After transfecting with control or YTHDF2 plasmid for 24 h, Act-D was added for the indicated times; furthermore the mature mRNA levels of KDM6B were checked in HeLa cells. Data are presented as mean ± SD from three independent experiments. *p < 0.05, **p < 0.01, ***p < 0.001, ns, no significant, by Student’s* t* test between two groups and by one-way ANOVA followed by Bonferroni test for multiple comparison

We then investigated the mechanisms responsible for m^6^A-regulated transcription of SLC7A11. It has been illustrated that m^6^A of chromosome-associated regulatory RNA regulates chromatin state and transcription [34, 35]. In general, transcription start sites of active genes are marked with trimethylated H3K4 (H3K4me3) or acetylated H3K27 (H3K27ac), and inactive genes are marked with H3K27me3 [35]. Next, we determined whether METTL3 affects histone modification marks (H3K27me3, H3K4me3, and H3K27ac) at the binding of SLC7A11 promoter. Our data showed that knockdown of METTL3 significantly decreased the binding of H3K27me3 to SLC7A11 promoter, while having no similar effect on the binding of H3K4me3 or H3K27ac, in HeLa cells (Fig. 3A). Similarly, the binding of H3K27me3 to SLC7A11 promoter in sh-METTL3 MDA-MB-231 cells was significantly decreased as compared with that in sh-control cells (Figure S3A). In addition, erastin treatment increased the binding of H3K27me3 to SLC7A11 promoter in both HeLa and MDA-MB-231 cells (Fig. 3B). These results indicated that METTL3 can regulate the H3K27 trimethylation of SLC7A11 promoter in cancer cells.

We then investigated the roles of H3K27 demethylases KDM6A and KDM6B in METTL3-regulated H3K27 trimethylation of the SLC7A11 promoter. Our data showed that si-KDM6B increased the binding of H3K27me3 to SLC7A11 promoter in HeLa cells (Fig. 3C), while si-KDM6A had no similar effect (Figure S3B). We carried out ChIP-qPCR assays with the H3K27me3 antibody, using non-specific IgG as a negative control and Histone H3 antibody as a positive control (Figure S3C). Further, METTL3 knockdown reversed the increase in the binding of H3K27me3 to the SLC7A11 promoter caused by si-KDM6B in HeLa (Fig. 3D) and MDA-MB-231 (Fig. 3E) cells. METTL3 KD can eliminate si-KDM6B-reduced the mRNA (Fig. 3F) and protein (Fig. 3G) expression of SLC7A11 in HeLa cells. Consistently, sh-METTL3 KD can eliminate si-KDM6B-reduced the mRNA (Figure S3 D) and protein (Figure S3E) expression of SLC7A11 in MDA-MB-231 cells. We further used GSK-J4 to inhibit KDM6B and investigated the causal regulation of METTL3 on transcription of SLC7A11. The results showed that GSK-J4 suppressed the mRNA (Figure S3F) and protein (Figure S3G) expression of SLC7A11. Moreover, our data showed that the expression of KDM6B was increased, while the levels of H3K27me3 were decreased, in METTL3 KD HeLa cells (Fig. 3H). These findings indicated that METTL3 negatively regulated the expression of KDM6B to modulate the H3K27 trimethylation of the SLC7A11 promoter.

Our data confirmed that KDM6B mRNA is m^6^A methylated, showing significant enrichment of m^6^A in its CDS regions (Fig. 3I), while KDM6A, EZH1, and EZH2 did not exhibit such modifications (Figure S3H), consistent with published reports in THP-1 cells [35]. Further, SRAMP predictions suggested that there were two areas with very high confidence of m^6^A at KDM6B mRNA (Figure S3I), the latter one matching the m^6^A sequencing data. m^6^A-RIP-qPCR confirmed that a sixfold increase in m^6^A antibody enriched KDM6B mRNA in HeLa cells, while this enrichment was significantly decreased in METTL3 KD HeLa cells (Fig. 3J). Consistently, knockdown of METTL3 significantly attenuated the enrichment of the m^6^A antibody on KDM6B mRNA in MDA-MB-231 cells (Fig. 3K). Previous studies indicated that YTHDF2 binds and decays the KDM6B transcripts via an m^6^A-dependent mechanism [35]. Our data showed that the mRNA stability of KDM6B in METTL3 knockdown HeLa cells was significantly greater than that in control HeLa cells (Fig. 3L). Consistently, decreased half-life of KDM6B mRNA was observed in sh-METTL3 MDA-MB-231 cells as compared with that in sh-control cells (Figure S3J). RIP-PCR analysis showed that YTHDF2 was remarkably enriched in KDM6B mRNA, while this relative enrichment was significantly suppressed in METTL3 KD cells (Fig. 3M). Overexpression of YTHDF2 significantly decreased the mRNA and protein expression of KDM6B in HeLa cells (Fig. 3N). This is likely because the overexpression of YTHDF2 decreased the mRNA stability of KDM6B in cancer cells (Fig. 3O). These data indicated that METTL3 can negatively regulate the expression of KDM6B to modulate the H3K27 trimethylation of the SLC7A11 promoter.

### GATA3 was involved in METTL3-regulated transcription of SLC7A11

**Fig. 4:**
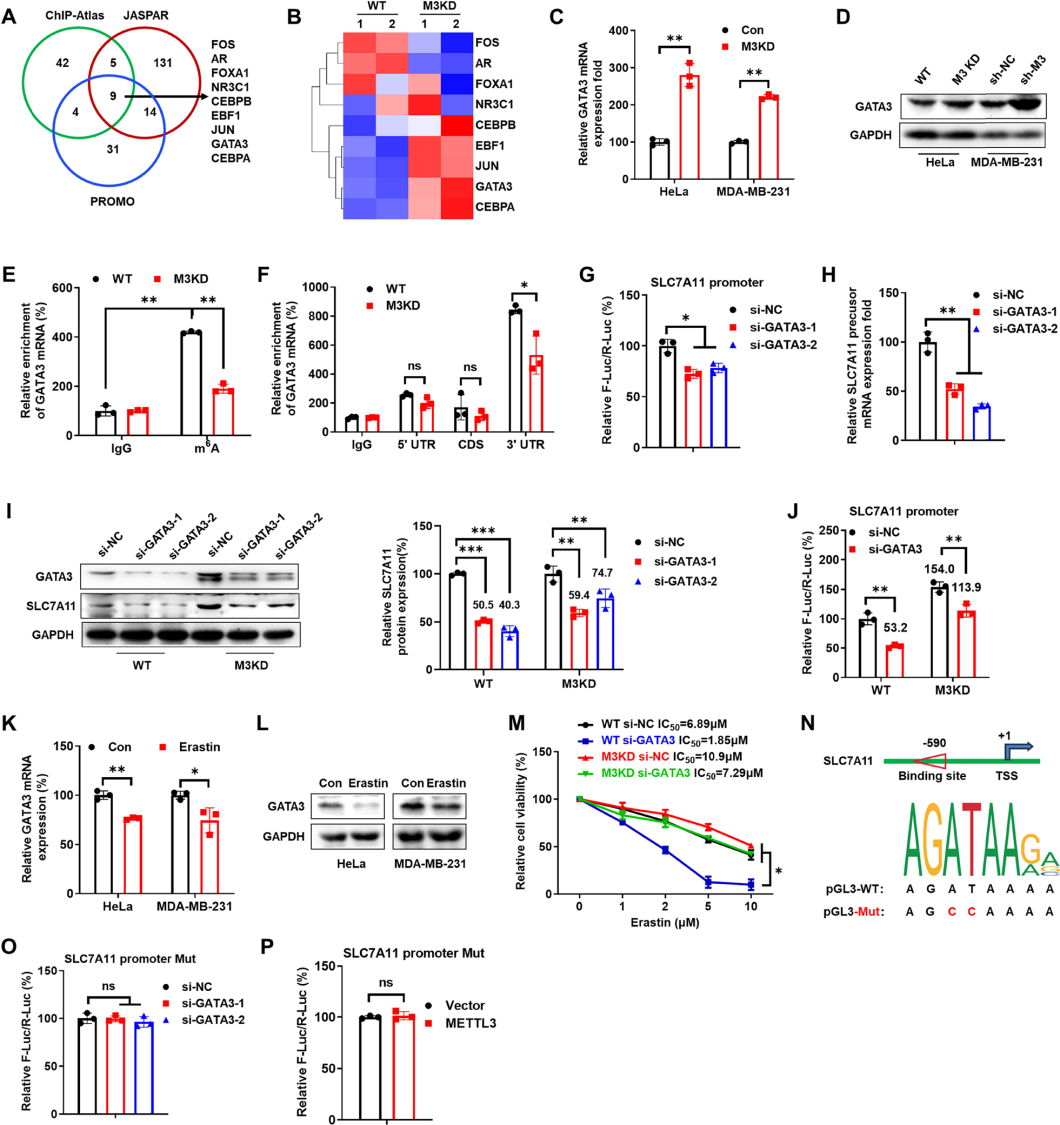
GATA3 was involved in METTL3-regulated transcription of SLC7A11. **A** The venn diagram showed the overlap of transcription factors of SLC7A11 predicted by PROMO, JASPAR, ChIPBase and ChIP-Atlas, respectively. **B** Heatmap of SLC7A11 predicted transcription factors. **C** The expression of GATA3 mRNA was checked by qRT-PCR in HeLa and MDA-MB-231 cells. **D** The expression of GATA3 protein was checked by western blot analysis in HeLa and MDA-MB-231 cells. **E** m^6^A RIP-qPCR analysis of GATA3 mRNA in HeLa WT and METTL3 KD cells. **F** The m^6^A in 5′UTR, CDS and 3′UTR of GATA3 in WT or METTL3 KD HeLa cells was analyzed by m^6^A-RIP-qPCR using fragmented RNA. **G** HeLa cells were transfected with siRNAs of GATA3, pGL3-SLC7A11-WT promoter and pRL-TK plasmid for 48 h, then the luciferase activity of F-Luc/R-Luc was detected using Dual-Lumi™ II Luciferase Assay Kit. **H** HeLa cells were transfected with siRNAs of GATA3 for 24 h, and SLC7A11 precursor mRNA expression was checked by qRT-PCR. **I** HeLa cells were transfected with siRNAs of GATA3 for 48 h, and GATA3 and SLC7A11 protein expression were checked by western blot analysis (*left*) and quantitatively analyzed (*right*). **J** HeLa cells were transfected with siRNAs of GATA3 for 24 h; they were co-transfected with pGL3-SLC7A11 promoter reporter and pRL-TK for another 24 h. The luciferase activity of F-Luc/R-Luc was detected using Dual-Lumi™ II Luciferase Assay Kit. **K** The expression of GATA3 mRNA was checked by qRT-PCR in HeLa and MDA-MB-231 cells after treating with erastin (5 µM) for 24 h. **L** The expression of GATA3 protein was checked by western blot analysis in HeLa and MDA-MB-231 cells after treating with erastin (5 µM) for 24 h. **M** HeLa cells were transfected with siRNAs of GATA3, and then treated with increasing concentrations of erastin for 48 h, and the relative cell viability was detected using Cell Counting Kit-8 kit. **N** Schematic representation of the mutated promoter in pGL3-Basic-SLC7A11 reporter. **O** HeLa cells were co-transfected with siRNAs of GATA3, pGL3-SLC7A11 mutant promoter and pRL-TK plasmid for 24 h, then the luciferase activity of F-Luc/R-Luc was detected using Dual-Lumi™ II Luciferase Assay Kit. **P** HeLa cells were co-transfected with pGL3-SLC7A11 mutant promoter reporter, pRL-TK and METTL3 plasmid for 24 h, and the luciferase activity of F-Luc/R-Luc was detected using Dual-Lumi™ II Luciferase Assay Kit. Data are presented as mean ± SD from three independent experiments. *p < 0.05, **p < 0.01, ***p < 0.001, ns, no significant, by Student’s* t* test between two groups and by one-way ANOVA followed by Bonferroni test for multiple comparison

Since METTL3 can negatively regulate the pGL-SLC7A11 promoter reporter, this suggests that transcription factors (TFs) may also be involved in m^6^A-regulated transcription of SLC7A11. To identify TFs involved in m^6^A regulated transcription of SLC7A11, we analyzed the potential transcription factors using ChIP-Atlas [24], PROMO with 1% maximum matrix dissimilarity rate [36], and JASPAR [37]. Among the top 60 factors identified by ChIP-Atlas, 159 factors identified by JASPAR, and 58 factors identified by PROMO (Table S2), nine factors including AR, CEBPA, CEBPB, EBF1, FOS, FOXA1, GATA3, JUN and NR3C1 were overlapped among the three databases (Fig. 4A). Among the identified potential TFs, varied mRNA levels of EBF1, JUN, GATA3 and CEBPA were observed in mRNA-seq data (Fig. 4B). Further, qPCR results showed that only GATA3, while not EBF1, JUN or CEBPA, was increased in both METTL3 KD HeLa and MDA-MB-231 cells (Fig. 4C, S4A). In addition, METTL3 knockdown increased the protein expression of GATA3 in HeLa and MDA-MB-231 cells (Fig. 4D). It indicated that METTL3 could negatively regulate the expression of GATA3 in cancer cells. m^6^A-RIP-qPCR confirmed that a fourfold enrichment of the m^6^A antibody was observed in GATA3 mRNA in HeLa cells, while this enrichment was significantly decreased in METTL3 KD HeLa cells (Fig. 4E). Consistently, knockdown of METTL3 significantly attenuated the m^6^A antibody enrichment of GATA3 mRNA in MDA-MB-231 cells (Figure S4B). Further, m^6^A-RIP analysis using fragmented RNA showed that GATA3 mRNA was modified by m^6^A and showed significant enrichment of m^6^A in its 3′UTR region (Fig. 4F), which is consistent with published reports using human HEK293T [38], A549 [39], and GM12878 [40] cells. It indicated that METTL3 can regulate the m^6^A methylation of GATA3 mRNA.

Promoter luciferase (Fig. 4G) and qRT-PCR for precursor mRNA analysis (Fig. 4H) confirmed that si-GATA3 decreased transcription of SLC7A11. This confirmed that GATA3 positively regulated the transcription of SLC7A11 in cancer cells. We further investigated whether GATA3 was involved in m^6^A-regulated expression of SLC7A11 and ferroptosis. Knockdown of GATA3 led to a decrease in the expression of SLC7A11 and significantly attenuated the METTL3 knockdown-induced upregulation of SLC7A11 in HeLa (Fig. 4I) and MDA-MB-231(Figure S4C) cells. Consistently, luciferase analysis confirmed that knockdown of GATA3 decreased the expression and significantly attenuated METTL3 knockdown-induced upregulation of SLC7A11 promoter activity (Fig. 4J). Further, erastin significantly decreased the mRNA (Fig. 4K) and protein (Fig. 4L) expression of GATA3 in both HeLa and MDA-MB-231 cells. Additionally, knockdown of GATA3 also increased the erastin sensitivity of both HeLa cells (Fig. 4M). All these data indicated that GATA3 was involved in the negative effects of METTL3 on transcription of SLC7A11 and ferroptosis activity of cancer cells.

According to the prediction results by JASPAR, there is one putative site with 90% similarity in the sequence of the GATA3 binding motif in the SLC7A11 promoter. We then mutated the putative site of the SLC7A11 promoter to generate the pGL3-SLC7A11-Mut reporter (Fig. 4N). Results showed that the mutation of the site can significantly abolish si-GATA3-induced reduction of F-Luc in HeLa cells (Fig. 4O). Further, METTL3 had significantly less effect on the pGL3-SLC7A11-Mut reporter in HeLa cells (Fig. 4P). All these data suggested that GATA3, which was regulated by METTL3, could regulate the transcription of SLC7A11 via binding to the putative site at − 597 to − 590 of SLC7A11 promoter.

### METTL3 decreased precursor mRNA stability of GATA3 via m^6^A dependent recruitment of Dis3L2

**Fig. 5:**
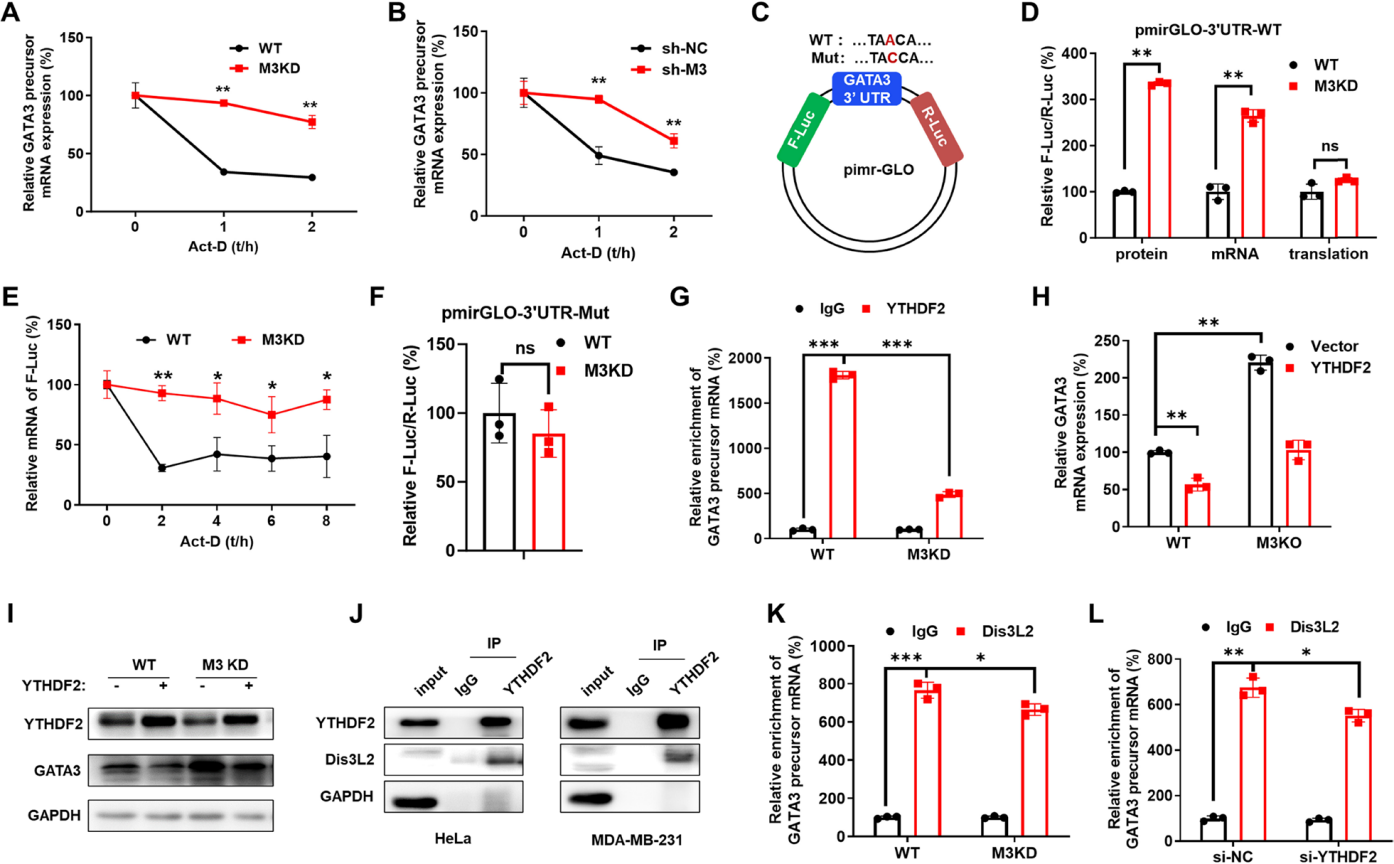
METTL3 decreased precursor mRNA stability of GATA3 via m^6^A dependent recruitment of Dis3L2. **A** After treatment with Act-D for the indicated times, the precursor mRNA levels of GATA3 were checked in WT and METTL3 KD HeLa cells by qRT-PCR. **B** After treatment with Act-D for the indicated times, the precursor mRNA levels of GATA3 were checked in sh-NC and sh-METTL3 MDA-MB-231 cells by qRT-PCR. **C** Schematic representation of the pmirGLO-GATA3-3′UTR reporter. **D** Wild-type or METTL3 KD HeLa cells were transfected with pmirGLO-GATA3-3′UTR reporter for 24 h. The translation outcome was determined as a relative signal of F-luc divided by R-luc, the mRNA abundance was determined by qRT-PCR of and R-luc, and the translation efficiency of GATA3 is defined as the quotient of reporter protein production (F-luc/R-luc) divided by mRNA abundance. **E** HeLa cells were transfected with pmirGLO-GATA3-3′UTR for 24 h and then further treated with Act-D for the indicated times. The mRNA of F-luc was checked by qRT-PCR. **F** HeLa cells were transfected with pmirGLO-GATA3-3′UTR-Mut for 24 h, and the relative luciferase activity of F-Luc/R-Luc was measured. **G** YTHDF2 RIP-qPCR analysis of GATA3 precursor mRNA in HeLa cells. **H** HeLa cells were transfected with vector or YTHDF2, and GATA3 mRNA expression was measured by qRT-PCR. **I** HeLa cells were transfected with vector or YTHDF2, and proteins expression were measured by western blot analysis. **J** Protein lysates of HeLa and MDA-MB-231 cells were immunoprecipitated with YTHDF2 antibodies. The binding between YTHDF2 and Dis3L2 protein was checked by immunoprecipitation. **K** Dis3L2 RIP-qPCR analysis of GATA3 precursor mRNA in HeLa wild type and METTL3 KD cells. **L** Dis3L2 RIP-qPCR analysis of GATA3 precursor mRNA in HeLa after transfecting with siRNA of YTHDF2 for 24 h. Data are presented as mean ± SD from three independent experiments. *p < 0.05, **p < 0.01, ***p < 0.001, ns, no significant, by Student’s* t* test between two groups and by one-way ANOVA followed by Bonferroni test for multiple comparison

We then investigated the potential mechanisms for METTL3-regulated expression of GATA3. Firstly, our data showed that knockdown of METTL3 had no effect on GATA3 protein stability (Figure S5 A). In addition, knockdown of METTL3 also had no effect on the stability of mature GATA3 mRNA (Figure S5B). However, knockdown of METTL3 increased the stability of precursor GATA3 mRNA in both HeLa (Fig. 5A) and MDA-MB-231 (Fig. 5B) cells. This indicated that METTL3 decreases the stability of precursor GATA3 mRNA to suppress the expression of GATA3.

The mechanisms underlying METTL3-mediated decrease in precursor GATA3 mRNA stability were further investigated. m^6^A-RIP-PCR showed that the m^6^A enrichment of GATA3 3′UTR was significantly down-regulated in METTL3 KD cells (Fig. 4F), suggesting that m^6^A methylation in 3′UTR regions is dynamic. The SRAMP prediction results suggested that there were high-confidence m^6^A sites at GATA3 precursor mRNA (Figure S5C). Previous reports elucidated that the GATA3 3′UTR maintains the KIAA1429-mediated m^6^A regulation [41]; therefore, we focused on the m^6^A site in the 3′UTR of GATA3. To study the potential roles of m^6^A methylation on GATA3 expression, we generated luciferase reporters containing a firefly luciferase, followed by the wild type GATA3 3′UTR, or mutant 3′UTR (site 2144, “A” to “C”, Fig. 5C). The dual-luciferase assay showed that the mRNA expression (Fig. 5D) and mRNA stability (Fig. 5E) of pmirGLO-GATA3-3′UTR in METTL3 KD HeLa cells were significantly greater than that in wild-type HeLa cells. However, the mutation of the m^6^A peak in 3′UTR abolished these regulatory effects of METTL3 on the expression of pmirGLO-GATA3-3′UTR (Fig. 5F).

It has been reported that YTHDF2 is the only reader protein for m^6^A-induced decay of mRNA [42]. RIP-PCR analysis showed that YTHDF2 was enriched in GATA3 precursor mRNA remarkably, while this enrichment was significantly suppressed in METTL3 KD cells (Fig. 5G, and Figure S5D). Further, the overexpression of YTHDF2 significantly decreased the mRNA and protein expression of GATA3 in HeLa cells, while this effect was rescued in METTL3 knockdown HeLa cells (Fig. 5H and I). This indicated that YTHDF2 was responsible for m^6^A induced degradation of GATA3 mRNA in cancer cells.

It has been revealed that YTHDF2 can interact with deadenylase, exosomes and helicases to regulate RNA stability [43]. The 3′−5′ exoribonuclease Dis3L2 can bind with the 3′ polyuridine of RNA substrates to mediate the degradation of uridylated RNA [44]. Our data showed that YTHDF2 can directly bind to Dis3L2 in both HeLa and MDA-MB-231 cells (Fig. 5J). Further, RIP-PCR assay showed that Dis3L2 can directly bind with the precursor mRNA of GATA3, while the binding was decreased in METTL3 knockdown HeLa cells (Fig. 5K). To investigate whether YTHDF2 was essential for Dis3L2-induced degradation of GATA3, its expression was silenced using siRNA (Figure S5 E). The results showed that si-YTHDF2 significantly decreased the binding between Dis3L2 and GATA3 mRNA (Fig. 5L). The data suggested that YTHDF2 binds with m^6^A methylated GATA3 mRNA and recruits Dis3L2 to induce its degradation in cancer cells.

### YY1 was responsible for the erastin induced-upregulation of METTL3

**Fig. 6:**
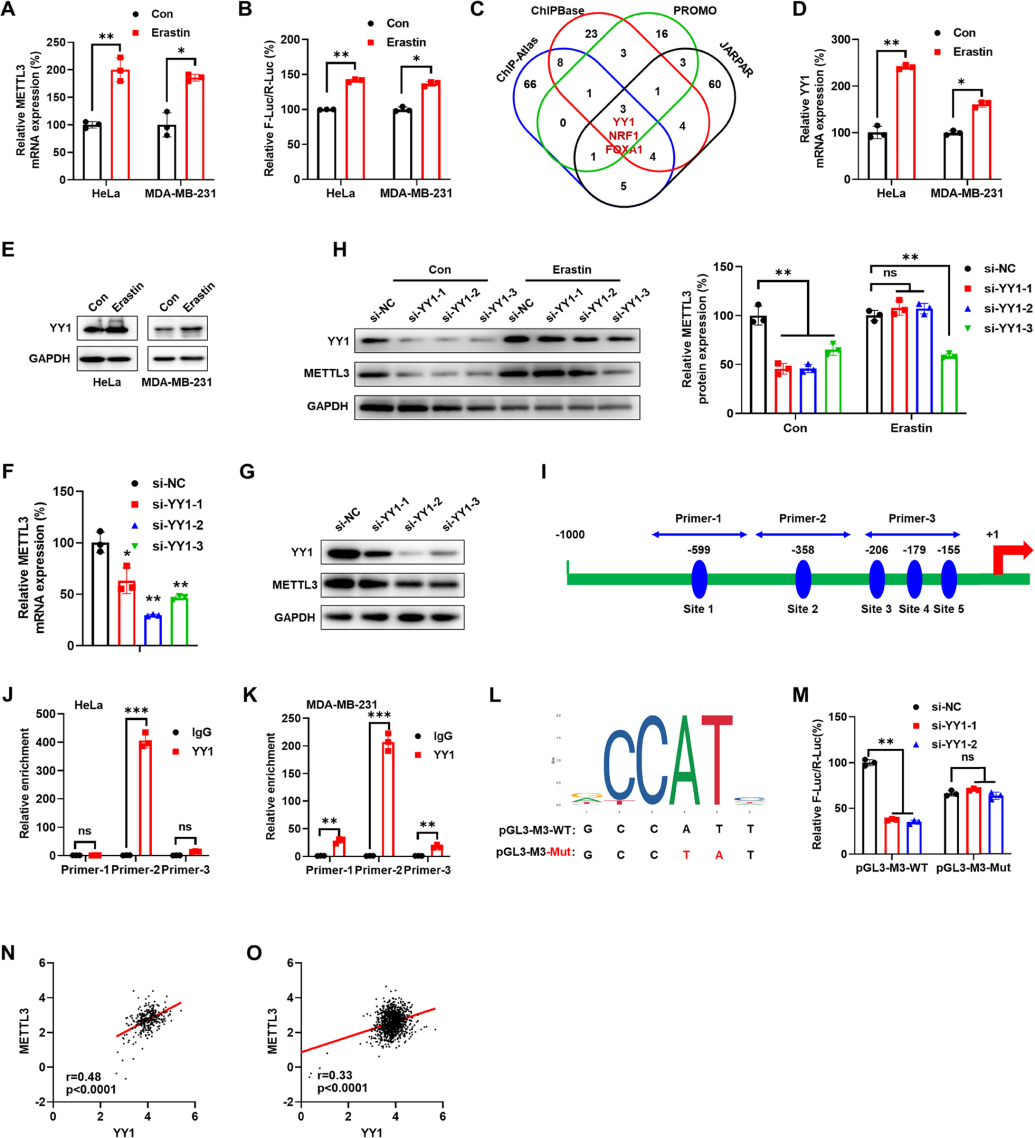
YY1 was responsible for the erastin induced-upregulation of METTL3. **A** The expression of METTL3 mRNA was checked by qRT-PCR in HeLa and MDA-MB-231 cells after treating with erastin (2 µM) for 24 h. **B** The relative luciferase activity of F-Luc/R-Luc of pGL3-Basic-METTL3 promoter after treating with erastin (2 µM) for 24 h. **C** Venn diagram showed the overlap of transcription factors of METTL3 predicted by PROMO, JASPAR, ChIPBase and ChIP-Atlas, respectively. **D** The expression of YY1 mRNA was checked by qRT-PCR in HeLa and MDA-MB-231 cells after treating with erastin (2 µM) for 24 h. **E** The expression of YY1 protein was checked by western blot analysis in HeLa and MDA-MB-231 cells after treating with erastin (2 µM) for 24 h. **F** The expression of YY1 mRNA was checked by qRT-PCR in HeLa after transfecting with siRNAs of YY1 for 24 h. **G** The expression of YY1 protein was checked by western blot analysis in HeLa after transfecting with siRNAs of YY1 for 48 h. **H** The expression of YY1 protein was checked by western blot analysis (*left*) and quantitatively analyzed (*right*) in HeLa after transfecting with erastin and siRNAs of YY1 for 48 h. **I** Binding sites of YY1 and primer for ChIP-qPCR to the METTL3 promoter region. **J** ChIP-qPCR assays to examine YY1 binding to the METTL3 promoter in HeLa cells. **K** ChIP-qPCR assays to examine YY1 binding to the METTL3 promoter in MDA-MB-231 cells. **L** Schematic representation of the mutant promoter in pGL3-Basic-METTL3 reporter. **M** HeLa cells were co-transfected with pGL3-METTL3-WT-Luc, pGL3-METTL3-Mut-Luc, pRL-TK plasmid and si-NC or si-YY1-1 and si-YY1-2 for 48 h. Results were presented as the ratio between the activity of the reporter plasmid and pRL-TK. **N** Correlation between METTL3 and YY1 in cervical cancer patients (n = 306) from TCGA. **O** Correlation between METTL3 and YY1 in breast cancer patients (n = 1085) from TCGA. Data are presented as mean ± SD from three independent experiments. *p < 0.05, **p < 0.01, ***p < 0.001, ns, no significant, by Student’s* t* test between two groups and by one-way ANOVA followed by Bonferroni test for multiple comparison

We evaluated the potential mechanisms responsible for erastin-regulated expression of METTL3 in cancer cells. Our data showed that erastin could induce the precursor and mature mRNA expression of METTL3 in both HeLa and MDA-MB-231 cells (Fig. 6A), while it had no effect on the mRNA stability of METTL3 (Figure S6A). Further, we generated the promoter reporter of METTL3 by inserting 1 kb upstream of the transcription start site (TSS) of METTL3 into the pGL3 plasmid. The luciferase assay showed that the promoter activities of METT3 in cancer cells treated with erastin were significantly greater than those in control cells (Fig. 6B). This indicated that the transcription of METTL3 was activated in cancer cells.

To identify TFs involved in erastin-regulated transcription of METTL3, we analyzed the potential transcription factors using ChIP-Atlas [24], PROMO with a 1% maximum matrix dissimilarity rate [36], JASPAR [37], and ChIPBase. Among the top 88 factors identified by ChIP-Atlas, 83 factors identified by JASPAR, 28 factors identified by PROMO, and 47 factors identified by ChIPBase, three factors including YY1, NRF1 and FOXA1, were overlapping among all databases (Fig. 6C, Table S3). We further checked the effect of erastin on the expression of these genes, and only the mRNA of YY1 was upregulated in erastin-induced cancer cells (Fig. 6D, and Figure S6B). Our data showed that erastin can induce the protein expression of YY1 in HeLa and MDA-MB-231 cells (Fig. 6E). We then knocked down the expression of YY1 using its specific siRNA (Figure S6C). Our data showed that knockdown of YY1 suppressed the mRNA (Fig. 6F) and protein (Fig. 6G) expression of METTL3 in HeLa cells. Further, knockdown of YY1 markedly attenuated erastin-induced expression of METTL3 in both HeLa (Fig. 6H) and MDA-MB-231(Figure S6D) cells. These data indicated that YY1 was involved in erastin induced expression of METTL3 in cancer cells.

JASPAR showed that there were 5 putative sites with 94% similarity in the sequence of YY1 binding motif to the promoter within 1 kb upstream of METTL3. Three pairs of primers were designed for the five main regions with a dense distribution of this motif, among which primer-3 containing three binding sites (Fig. 6I). ChIP-qPCR showed that binding of YY1 to the potential binding site 2 was much greater than to the other sites in HeLa (Fig. 6J) and MDA-MB-231 (Fig. 6K) cells, which was consistent with previous reports [45]. Then the potential binding site 2 of the METTL3 promoter was mutated (Fig. 6L). Our data showed that si-YY1 significantly decreased the luciferase activity of pGL3-METTL3-WT, while the inhibitory effect of si-YY1 was attenuated for pGL3-METTL3-Mut (Fig. 6M). Further, the expression of YY1 was significantly positively correlated with the expression of METTL3 in clinical cervical (Fig. 6N) and breast (Fig. 6O) cancer patients from TCGA. All these data suggested that YY1 might be responsible for erastin-induced transcription of METTL3 via binding to its promoter-proximal site.

### Targeting m^6^A/METTL3 repressed SLC7A11 expression

**Fig. 7:**
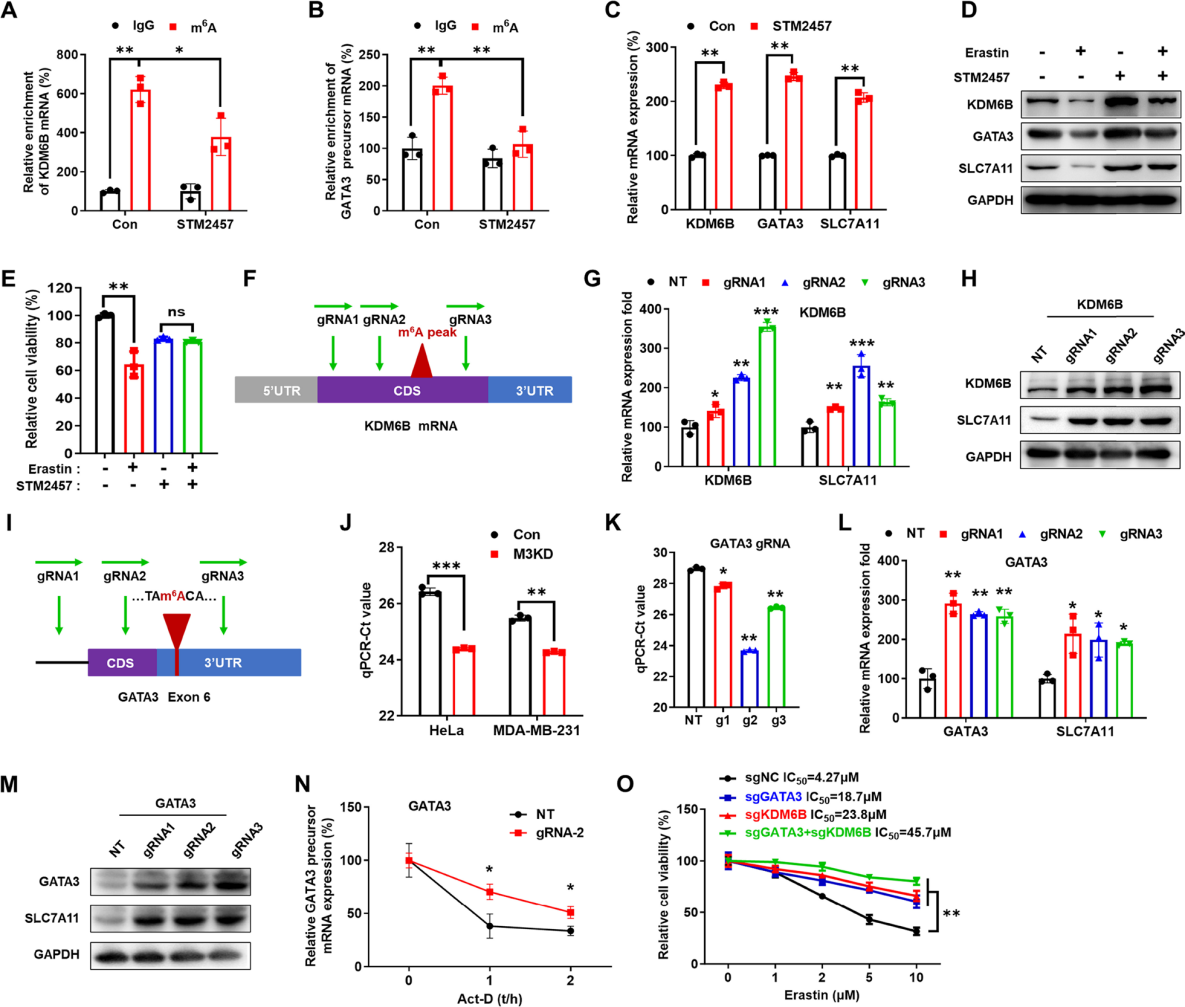
Targeting m^6^A/METTL3 repressed SLC7A11 expression. **A** m^6^A RIP-qPCR analysis of KDM6B mRNA in HeLa cells treated with or without STM2457(5 μM) for 24 h. **B** m^6^A RIP-qPCR analysis of GATA3 precursor mRNA in HeLa cells treated with or without STM2457(5 μM) for 24 h. **C** The KDM6B, GATA3 and SLC7A11 mRNA expression levels were measured by qRT-PCR in HeLa cells treated with or without STM2457 (5 μM) for 24 h. **D** The KDM6B, GATA3 and SLC7A11 protein expression levels were measured by western blot analysis in HeLa cells treated with or without STM2457 (5 μM) and erastin (2 μM) for 24 h. **E** The cell viability was measured after with or without STM2457 (5 μM) and erastin (2 μM) for 24 h. **F** Schematic representation of positions of m^6^A peaks within KDM6B mRNA and the regions targeted by three gRNAs, respectively. **G** The KDM6B and SLC7A11 mRNA expression levels in HeLa cells transfected with dCas13b-ALKBH5 combined with gRNAs for 24 h. **H** The KDM6B and SLC7A11 protein expression levels in HeLa cells transfected with dCas13b-ALKBH5 combined with gRNAs for 24 h. **I** Schematic representation of positions of m^6^A site within GATA3 precursor mRNA and the regions targeted by three gRNAs, respectively; **J** The threshold cycle (Ct) of qPCR showing SELECT results for detecting m^6^A site in the potential m^6^A site of GATA3 3′UTR in METTL3 KD HeLa, sh-METTL3 MDA-MB-231 and their corresponding control cells. **K** The threshold cycle (Ct) of qPCR showing SELECT results for detecting m^6^A site in the potential m^6^A site of GATA3 3′UTR in HeLa cells after transfecting with gRNAs and dCas13b-ALKBH5. **L** The GATA3 and SLC7A11 mRNA expression levels in HeLa cells transfected with dCas13b-ALKBH5 combined with gRNAs for 24 h. **M** The GATA3 and SLC7A11 protein expression levels in HeLa cells transfected with dCas13b-ALKBH5 combined with gRNA control or gRNA for 24 h. **N** HeLa cells were transfected with gRNA control, gRNA for GATA3, and dCas13b-ALKBH5 for 24 h and then further treated with Act-D for the indicated times. The precursor mRNA of GATA3 was checked by qRT-PCR. **O** HeLa cells were transfected with gRNA control, gRNA for GATA3, gRNA for KDM6B, and dCas13b-ALKBH5 for 24 h, and then further treated with increasing concentration of erastin for 48 h. The cell viability was measures after by Cell Counting Kit-8 kit. Data are presented as mean ± SD from three independent experiments. *p < 0.05, **p < 0.01, ***p < 0.001, ns, no significant, by Student’s* t* test between two groups and by one-way ANOVA followed by Bonferroni test for multiple comparison

Firstly, we evaluated the potential roles of STM2457, a highly potent and selective inhibitor of METTL3/14 [46], in ferroptosis and the expression of SLC7A11. Results showed that STM2457 significantly decreased the m^6^A enrichment of KDM6B (Fig. 7A) and GATA3 (Fig. 7B) in HeLa cells. Further, STM2457 increased the mRNA (Fig. 7C) and protein (Fig. 7D) expression of KDM6B, GATA3 and SLC7A11 in HeLa cells. STM2457 also reversed the erastin-induced reduction in KDM6B, GATA3, and SLC7A11 expression (Fig. 7D). In addition, STM2457 decreased the erastin sensitivity of both HeLa cells (Fig. 7E). These findings confirmed that targeting m^6^A via an METTL3 inhibitor can suppress erastin induced ferroptosis.

We then specifically demethylated the m^6^A of specific mRNA by fusing the catalytically dead Type VI-B Cas13 enzyme from *Prevotella* sp. P5-125 (dPspCas13b) with the m^6^A demethylase ALKBH5, which was developed in our lab and named dm^6^ACRISPR [47]. Firstly, the mRNA of KDM6B was targeted by three gRNAs at distinct positions, around the m^6^A peak (Fig. 7F). To test the efficiency of the gRNAs, we checked the mRNA expression of KDM6B in cells transfected with three gRNAs for KDM6B and wild type Cas13b, which cleaves the targeted mRNA. Our data showed that all three gRNAs combined with wild type Cas13b significantly decreased the mRNA levels of KDM6B (Figure S7A), suggesting that all three gRNAs combined with wild type of Cas13b can work efficiently. While transfection of gRNAs alone (Figure S7B) or gRNAs combined with dCas13b (Figure S7C) had no effect on mRNA expression of KDM6B. The dCas13b-ALKBH5 mediated demethylation of KDM6B was further confirmed by m^6^A-RIP-qPCR (Figure S7D). Our data showed that gRNAs of KDM6B and dCas13b-ALKBH5 transfection led to a significant upregulation of KDM6B and SLC7A11 in mRNA (Fig. 7G) and protein (Fig. 7H), suggesting that the demethylation of KDM6B mRNA can affect the expression of KDM6B and SLC7A11.

Similarly, we specifically demethylated the m^6^A of GATA3 precursor mRNA using dCas13b-ALKBH5, in which the nuclear export sequence (NES) was replaced by nuclear location sequence (NLS). Then the precursor mRNA of GATA3 was targeted by three gRNAs at distinct positions, around the m^6^A site (Fig. 7I). Firstly, the m^6^A site of GATA3 was verified by “SELECT” method (Xiao et al., 2018) in HeLa and MDA-MB-231 cells (Fig. 7J). Next, wild-type Cas13b was used to test the efficiency of gRNAs. Our data showed that all three gRNAs combined with wild-type Cas13b significantly decreased the mRNA levels of GATA3 (Figure S7E), suggesting that all three gRNAs combined with wild-type Cas13b can work efficiently. While transfection of gRNAs alone (Figure  S 7F) or gRNAs combined with dCas13b (Figure S7G) had no effect on mRNA expression of GATA3. The dCas13b-ALKBH5 mediated demethylation of GATA3 was further confirmed by m^6^A-RIP-qPCR (Figure S7H). We then checked the effect of gRNAs of GATA3 and dCas13b-ALKBH5 on m^6^A modification of GATA3. SELECT-qPCR showed that all three gRNAs combined with dCas13b-ALKBH5 significantly decreased the m^6^A levels of the targeted site (Fig. 7K).

Consistently, our data showed that gRNAs of GATA3 and dCas13b-ALKBH5 transfection led to a significant upregulation of GATA3 and SLC7A11 at the mRNA (Fig. 7L) and protein (Fig. 7M) levels. This might be because dm^6^ACRISPR with gRNA for GATA3 can significantly decrease the binding of GATA3 mRNA with YTHDF2 and Dis3L2 (Figure S7I). To investigate whether dCas13b-ALKBH5-induced upregulation of GATA3 was due to m^6^A-mediated mRNA stability, we compared the effects of dCAS13b-ALKBH5 or dCAS13b-ALKBH5 with gRNA2 on the half-life of GATA3 precursor mRNA. Results showed that targeted demethylation of GATA3 can significantly stabilize its precursor mRNA (Fig. 7N), suggesting that dm^6^ACRISPR can increase precursor mRNA stability through demethylation of m^6^A at the 3′UTR in the case of GATA3. Further, gRNA of KDM6B and GATA3 combined with dCas13b-ALKBH5 can significantly decrease the sensitivity of HeLa cells to erastin-induced ferroptosis (Fig. 7O). These data suggested that targeting m^6^A of KDM6B and GATA3, the transcriptional regulators of SLC7A11, by dm^6^ACRISPR affects ferroptosis of cancer cells.

### METTL3/SLC7A11 axis was involved in m^6^A regulated cancer progression

**Fig. 8:**
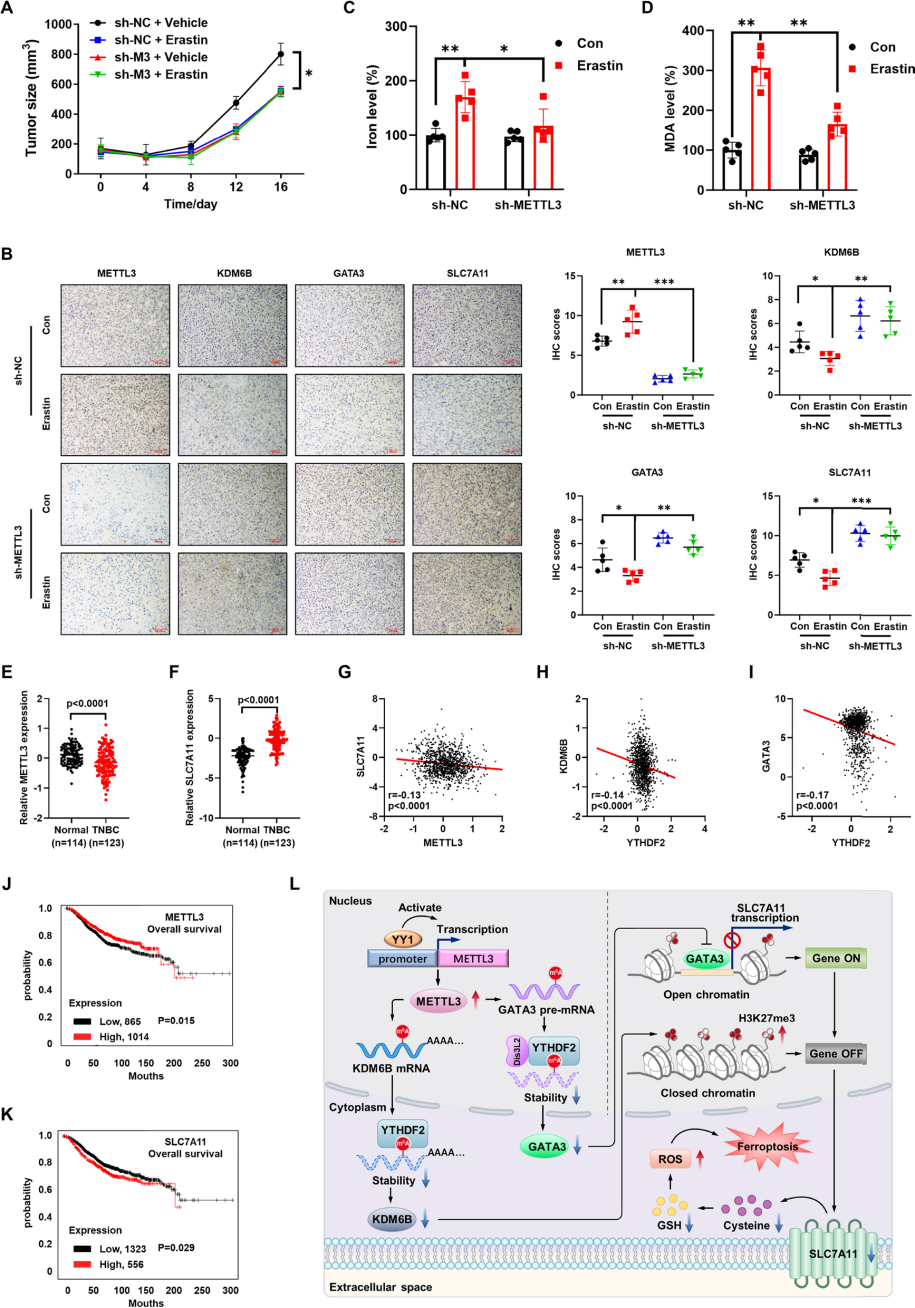
METTL3/SLC7A11 axis was involved in m^6^A regulated cancer progression. **A** Tumor growth curves of mice were xenografts implanted with sh-NC and sh-METTL3 MDA-MB-231 cells, further treated with vehicle or erastin at indicated time intervals. **B** IHC staining (*left*) and corresponding quantification (*right*) of paraffin-embedded sections obtained from a xenograft mouse model. n = 5, Scale bars: 100 μm. **C** Iron accumulation was analyzed using the Iron Assay Kit in the MDA-MB-231 xenograft mouse model. **D** MDA was analyzed using the MDA Assay Kit in the MDA-MB-231 xenograft mouse model. **E** METTL3 expression in breast cancer patients from the TCGA database. **F** SLC7A11 expression in breast cancer patients from the TCGA database. **G** Correlation between METTL3 and SLC7A11 in breast cancer patients (n = 1104) from the TCGA database. **H** Correlation between YTHDF2 and KDM6B in breast cancer patients (n = 1104) from the TCGA database. **I** Correlation between YTHDF2 and GATA3 in breast cancer patients (n = 1104) from the TCGA database. **J**, **K** The Kaplan–Meier survival curves of METTL3 and SLC7A11 in breast cancer patients from the TCGA database. **L** Schematic representation of the role of METTL3/SLC7A11 in the regulation of ferroptosis in cancer cells

We further investigated the potential roles of the METTL3/SLC7A11 axis in vivo breast cancer progression. To test the in vivo effects of METTL3/SLC7A11-mediated ferroptosis sensitivity, we implanted mice with sh-NC or sh-METTL3 MDA-MB-231 cells and further treated them with vehicle or erastin. Results showed that erastin treatment significantly inhibited the growth of MDA-MB-231 xenografts (Fig. 8A), and no significant change in body weight was observed among all groups (Figure S8A). Additionally, immunohistochemistry (IHC) data showed that sh-METTL3 increased the expression of KDM6B, GATA3 and SLC7A11 in the xenograft model, and sh-METTL3 attenuated erastin-induced downregulation of KDM6B, GATA3 and SLC7A11 (Fig. 8B). Further, we observed that sh-METTL3 evidently damaged erastin-induced redox-active iron accumulation in Fig. 8C, which indicated sh-METTL3 attenuated erastin-induced decrease in tumor size associated with a reduction in iron overload. Similarly, sh-METTL3 attenuated erastin-induced MDA (Fig. 8D), indicating that METTL3/SLC7A11 regulated the in vivo ferroptosis of xenografts.

We then analyzed the expression of the axis components and their correlation with clinical characteristics of breast cancer (BC). The expression of METTL3 in normal tissue was significantly lower than that in triple negative breast cancer (TNBC) according to The Cancer Genome Atlas (TCGA) database (Fig. 8E). While the expression of SLC7A11 in tumor tissue was significantly greater than that in normal tissue according to the TCGA database (Fig. 8F). Expression of METTL3 in estrogen receptor α (ERα) and progesterone receptor (PR) negative BCs was significantly (p < 0.05) lower than that in their corresponding positive BCs (Figure S8B), while the expression of SLC7A11 in ERRα and PR negative BCs was significantly (p < 0.05) greater than that in their corresponding-positive BCs (Figure S8C). In addition, the expression of METTL3 was decreased (Figure S8 D), while SLC7A11 was increased (Figure S8E), in TP53-positive BCs as compared with those in TP53-negative BCs.

The data from TCGA showed that the expression of SLC7A11 was negatively correlated (p < 0.0001) with the expression of METTL3 in BC patients (Fig. 8G), suggesting an in vivo correlation of the METTL3/SLC7A11 axis with disease progression. In addition, the expression of KDM6B and GATA3 was negatively correlated (p < 0.0001) with the expression of YTHDF2 in 1104 BC patients (Fig. 8H, I), indicating the role of YTHDF2 in the regulation of GATA3 and KDM6B. Using the online bioinformatics tool Kaplan–Meier plotter [48], we found that BC patients with increased expression of METTL3 had significantly increased OS (Fig. 8J). Furthermore, BC patients with increased expression of SLC7A11 showed significantly reduced OS (Fig. 8K). Altogether, these results suggest that the METTL3/SLC7A11 axis is positively associated with the clinical progression of BC.

## Discussion

As a newly discovered iron-dependent lipid peroxidation cell death pathway, ferroptosis is emerging as a therapeutic target for various cancers. Numerous oncogenic and tumor suppressor signaling pathways have been reported to be associated with ferroptosis [49]. In our study, we selected several representative cell lines: A549 (lung cancer), HeLa and SiHa (cervical cancer), and MDA-MB-231 (breast cancer). These cell lines were chosen to provide a comprehensive perspective on how m^6^A modification may regulate ferroptosis across different cancer contexts.

m^6^A regulates various ferroptosis-associated pathological processes, including aortic dissection [50], sepsis-associated acute lung injury [51] and various cancers [52, 53]. For example, total levels of m^6^A were evidently increased upon exposure to ferroptosis-inducing compounds due to the upregulation of METTL4 and the downregulation of FTO in hepatic stellate cells [28]. We found that sh-METTL4 can attenuate erastin-induced redox-active iron accumulation, while erastin does not affect the expression of METTL4. Therefore, we believe that although METTL4 is related to ferroptosis, it does not play a role in erastin-induced ferroptosis in cancer cells, which differs from its role in hepatic stellate cells. Our present study reveals that the level of mRNA m^6^A and expression of METTL3 are increased in erastin-induced ferroptosis, while knockdown/inhibition of METTL3 attenuated erastin-induced ferroptosis. Further, m^6^A/METTL3 modulates ferroptosis-associated cancer progression through the regulation of SLC7A11 transcription, as illustrated in Fig. 8L. This suggests that activation of METTL3 may be a potential approach to increase the sensitivity of ferroptosis-based therapy for cancer treatment.

SLC7A11 is an essential subunit of System Xc- that is involved in protecting cells from oxidative injury and lipid peroxide-induced ferroptosis [54]. Recent studies also indicate that SLC7A11 can be directly methylated by m^6^A to modulate its mRNA stability [18, 21], splicing [55], or degradation [22]. m^6^A methylation 5′UTR and 3′UTR regions regulate mRNA degradation of SLC7A11 in cancer cells [21, 22], while there is no m^6^A modification in SLC7A11 mRNA according to our data. This suggests that the regulatory mechanism for SLC7A11 may be cell and cancer type dependent. Further, we illustrate that METTL3 can suppress the transcription of SLC7A11 by enhancing H3K27 trimethylation enrichment at its promoter. Mechanistically, METTL3 induces H3K27 trimethylation of the SLC7A11 promoter by suppressing the mRNA stability of H3K27 demethylases KDM6B. It has been reported that METTL14 can bind H3K27me3 and recruit KDM6B to induce H3K27me3 demethylation, while depletion of METTL14 leads to a global increase in H3K27me3 levels along with a global gene suppression [56]. In addition, m^6^A demethylation by METTL3 depletion or site-specific m^6^A demethylation of selected circRNAs elevates the levels of carRNAs and promotes an open chromatin state and downstream transcription [34]. RNA m^6^A regulates transcription via DNA demethylation and transposable element chromatin activation [57, 58]. All these data confirm that m^6^A may regulate gene transcription via the regulation of chromatin regulators.

Since METTL3 can negatively regulate the pGL-SLC7A11 promoter reporter, it indicates that transcription factors are also involved in m^6^A-regulated transcription of SLC7A11.We found that GATA3 is involved in METTL3-regulated expression of SLC7A11 and ferroptosis in cancer cells. Mechanistically, METTL3-induced m^6^A methylation of 3′UTR facilitates the decay of GATA3 precursor mRNA via YTHDF2-dependent recruitment of Dis3L2, subsequently suppressing the GATA3-induced transcription of SLC7A11. Consistently, a previous study indicated that KIAA1429 induces m^6^A methylation on the 3′UTR of GATA3 pre-mRNA, leading to the degradation of its mRNA [59]. GATA3 can induce the expression of numerous tumor-suppressive genes by binding to and activating their promoters [60]. Our data not only show that GATA3 directly binds to the promoter of SLC7A11 to activate its expression, but also indicate that the expression of GATA3 is regulated by RNA m^6^A methylation.

The expression of METTL3 is dysregulated in cancer through different mechanisms [61]. It has been reported that KDM1A-mediated upregulation of METTL3 ameliorates Alzheimer's disease [62]. The transcription factor ETS1 recruits P300 and WDR5, which separately mediates H3K27ac and H3K4me3 histone modification in the promoter of METTL3, inducing METTL3 transcription activation [63]. In our previous study, TBP was shown to be responsible for the upregulation of METTL3 in cervical cancer cells [30]. Here, we demonstrate that YY1 is responsible for the upregulation of METTL3 induced by erastin. YY1 is identified as a transcription factor of METTL3, capable of direct binding to the promoter of METTL3 and facilitating its transcription. Consistently, YY1 enhances METTL3 expression as a transcription factor in the human acute myeloid leukemia cell lines Kasumi-1 and THP-1 [45]. All these data suggest that YY1 is one of the key transcriptional factors that triggers the transcription of METTL3 in human cells.

Our results further reveal that targeted m^6^A/METTL3 can disturb erastin induced ferroptosis. Firstly, targeting m^6^A using a METTL3 inhibitor can suppress erastin-induced ferroptosis. Furthermore, specific demethylation of GATA3 and KDM6B transcripts can increase the expression of SLC7A11, leading to a decrease erastin sensitivity of cancer cells. To our knowledge, this is the first reported method for targeted demethylation of specific transcripts to artificially manipulate cell ferroptosis sensitivity. Moreover, both in vitro and in vivo data suggest that METTL3/SLC7A11 is involved in m^6^A regulated growth and erastin sensitivity of cancer cells. METTL3 KD increases the expression of SLC7A11, further suppressing the erastin sensitivity of tumor cells in vivo. Clinical analysis confirms the negative correlation between METTL3 and SLC7A11 in breast cancer tissues. Low expression of METTL3, while high expression of SLC7A11, reduces the survival rate of cancer patients. Our data demonstrate that erastin-induced ferroptosis is mediated through METTL3-mediated inhibition of SLC7A11 expression in vivo and in vitro.

In summary, we provide compelling in vitro and in vivo evidence demonstrating that m^6^A regulates the transcription of SLC7A11 through GATA3 and KDM6B to modulate ferroptosis. The methylation of GATA3 precursor mRNA and KDM6B mRNA is critical for m^6^A-mediated mRNA stability of GATA3 and KDM6B. While numerous genes are involved in ferroptosis, we cannot rule out the possibility that m^6^A modification indirectly targets other genes to regulate ferroptosis. In most cases, METTL3 has been reported as an oncogene that promotes the initiation and development of various cancers [61]. Our study suggests that METTL3 knockdown leads to the upregulation of SLC7A11, another potential oncogene, through GATA3 and KDM6B, thereby expanding our understanding of such interplays that are essential for therapeutic application.

## Conclusions

Our study reveals that METTL3 regulates the transcription of SLC7A11 through GATA3 and KDM6B to modulate ferroptosis in an m^6^A-dependent manner. This study expands the research on the mechanisms related to ferroptosis and provides a new potential strategy for the future treatment of cancer.

## Limitations of the study

We selected several representative cancer cell lines for our study, including one lung cancer cell line (A549), two cervical cancer cell lines (HeLa and SiHa), and one breast cancer cell line (MDA-MB-231). Given the limited number of cell lines used, the reliability of our conclusions for all tumors requires further validation. To support the universality of our findings, additional verification will be conducted with more cell lines and in vivo experiments.

## Supplementary Information

Additional file 1.

## Data Availability

The data that support the findings of this study are available from the corresponding author upon reasonable request.
