# Supplementary material for: N6-methyladenosine RNA modification regulates the transcription of SLC7A11 through KDM6B and GATA3 to modulate ferroptosis
Source: J Biomed Sci. 2025 Jan 13;32:8. doi: 10.1186/s12929-024-01100-y (PMC11726933; doi:10.1186/s12929-024-01100-y)
Supplement: Supplementary file 1 — Additional file 1. [file 12929_2024_1100_MOESM1_ESM.docx]

**Supplementary data for**

**N6-methyladenosine RNA modification regulates the transcription of SLC7A11 through KDM6B and GATA3 to modulate ferroptosis**

Zhang et al.

**
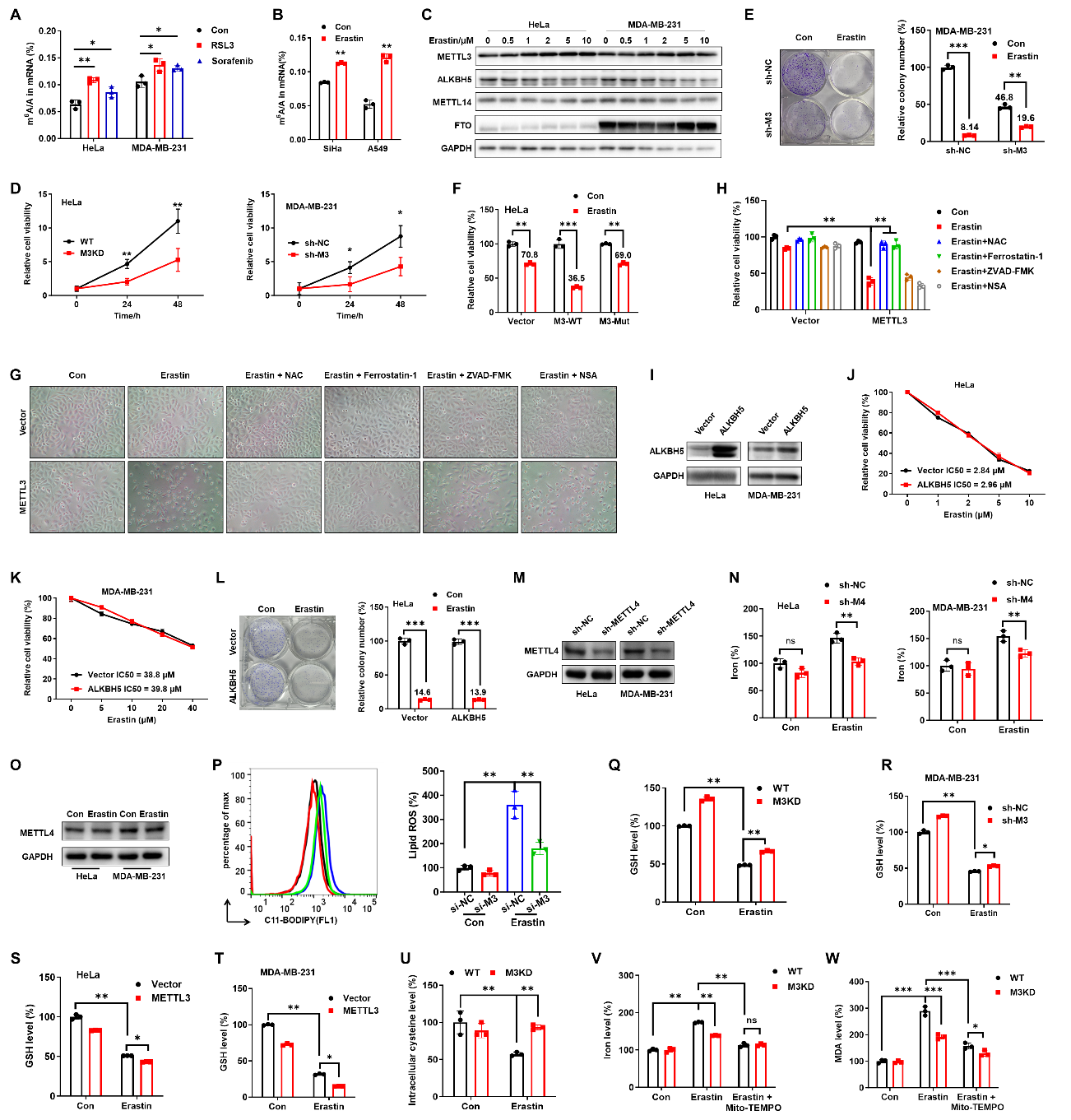
**

**Figure S1. METTL3 facilitated erastin-induced ferroptosis and enhanced mitochondrial ROS.**

1. HeLa and MDA-MB-231 cells were treated with or without RSL3 (1 μM) or sorafenib (2.5 μM) for 24 h, the m^6^A/A ratios of mRNA were checked by HPLC/MS/MS.
2. Cells were treated with or without 2 μM erastin for 24 h, the m^6^A/A ratios of mRNA were checked by LC/MS/MS.
3. HeLa and MDA-MB-231 cells were treated with increasing concentrations of erastin for 24 h, and the expression of m^6^A relative proteins were checked by western blot analysis.
4. The cell proliferation of HeLa and MDA-MB-231 cells were detected by Cell Counting kit-8 kit.
5. MDA-MB-231 cells were treated with or without erastin (0.1 μM) and Ferrostatin-1 (1 μM) for 14 days, the colonization capability was checked (*left*) and analyzed (*right*).
6. HeLa cells were transfected with vector control, METTL3 WT plasmid, or METTL3 DA mutant plasmid, and then the relative cell viability was detected using Cell Counting kit-8 kit after treating with or without erastin (2 μM) for 24h.

(G&H) Representative phase-contrast images (F) and quantitatively analysis (G) of METTL3 over expression HeLa cells treated with erastin (2 μM), ferrostatin-1 (1 μM), acetylcysteine (NAC, 2 mM), Z-VAD-FMK (10 μM) and necrosulfonamide (NSA, 5 μM) for 24 h

1. The expression of ALKBH5 in cells transfected with vector control or ALKBH5 plasmid.
2. HeLa cells were transfected with vector control or ALKBH5 plasmid, and then the relative cell viability was detected using Cell Counting Kit-8 after treating with increasing concentration of erastin for 48 h.
3. MDA-MB-231 cells were transfected with vector control or ALKBH5 plasmid, and then the relative cell viability was detected using Cell Counting Kit-8 after treating with increasing concentration of erastin for 48 h.
4. HeLa cells were transfected with vector control or ALKBH5 plasmid, treating with or without erastin (0.1 μM) and Ferrostatin-1 (1 μM) for 14 days. The colonization capability was checked (*left*) and analyzed (*right*).
5. The protein expression of METTL4 in HeLa and MDA-MB-231 cells was checked by western blot analysis.
6. Iron accumulation was analyzed using the Iron Assay Kit.
7. The protein expression of METTL4 in HeLa and MDA-MB-231 cells was checked by western blot analysis.
8. si-NC and si-METTL3 MDA-MB-231 cells were treated with or without erastin (2 μM) for 24 h, and lipid ROS production was assayed by flow cytometry using C11-BODIPY
9. The GSH levels in WT or METTL3 KD HeLa cells were detected after treating with or without erastin (2 μM) for 5 h.
10. The GSH levels in sh-NC or sh-METTL3 MDA-MB-231 cells were detected after treating with or without erastin (5 μM) for 5 h.
11. HeLa cells were transfected with vector control or METTL3 WT plasmid for 24h, and then the GSH levels in cells were detected after treating with or without erastin (2 μM) for 5 h.
12. MDA-MB-231 cells were transfected with vector control or METTL3 WT plasmid for 24h, and then the GSH levels in cells were detected after treating with or without erastin (5 μM) for 5 h.
13. WT or METTL3 KD HeLa cells were treated with or without erastin for (2 μM) for 12 h, and then the intracellular cysteine levels was detected

(V&W) HeLa cells were treated with or without erastin (2 μM) or Mito-TEMPO (1 μM) for 24 h: (V) Iron accumulation was analyzed using the Iron Assay Kit; (W) The intracellular MDA was detected by Microscale Malondialdehyde assay kit.

Data are presented as mean ± SD from three independent experiments. **p*<0.05, ***p*<0.01, ****p*<0.001, ns, no significant, by Student’s *t* test between two groups and by one-way ANOVA followed by Bonferroni test for multiple comparison.

**Related to Figure 1.**


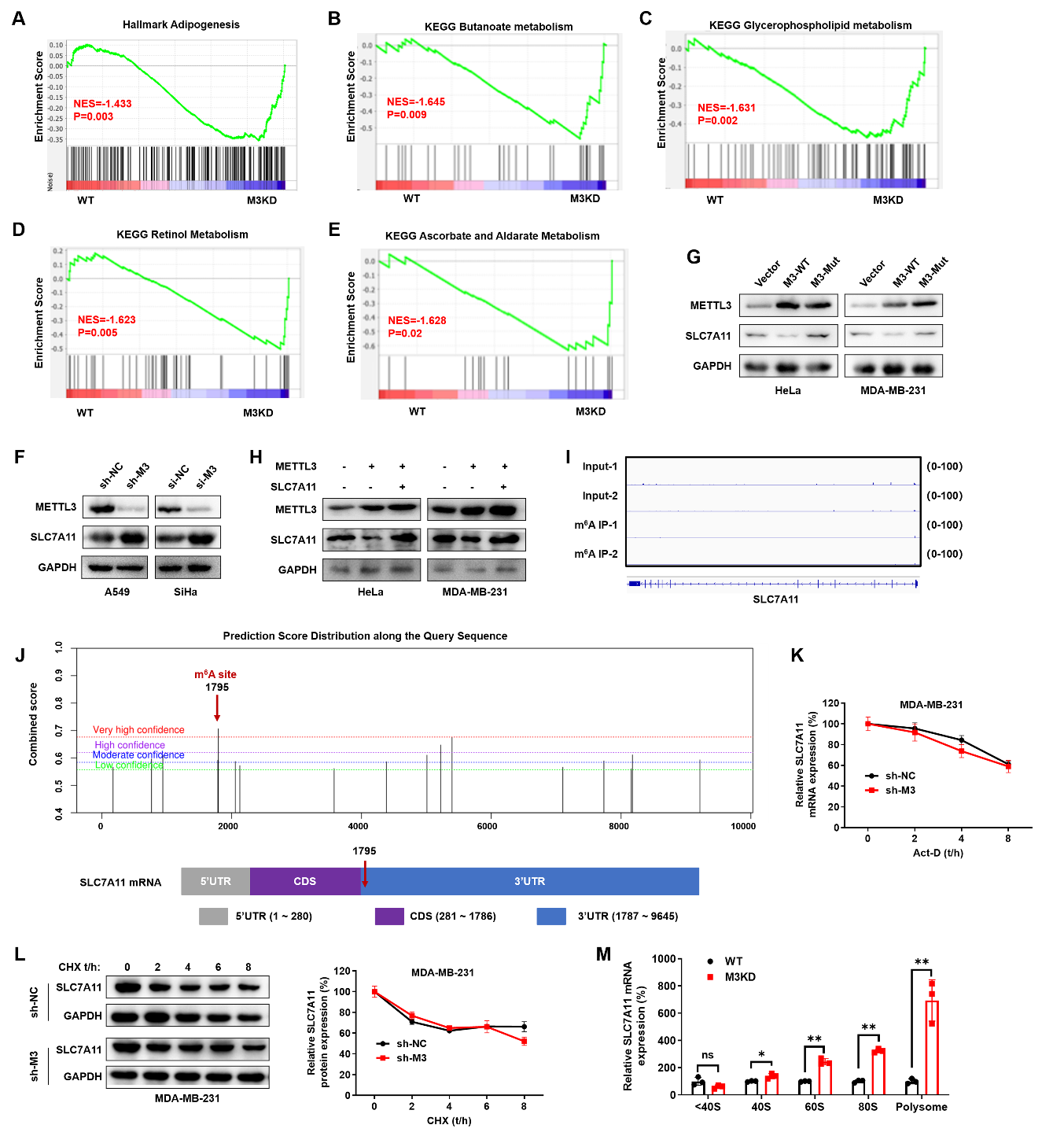
 **Figure S2. METTL3 triggered ferroptosis via suppressing SLC7A11 transcription.**

(A~E) GSEA reveals negative enrichment of genes in ferroptosis sets of METTL3 KD HeLa cells

1. The protein expression of SLC7A11 in A549 and SiHa cells was checked by western blot analysis.
2. HeLa and MDA-MB-231 cells were transfected with vector control, METTL3 plasmid or METTL3 DA mutant for 48 h, the protein expression of SLC7A11 and METTL3 was checked by western blot analysis.
3. HeLa and MDA-MB-231 cells were transfected with vector control, METTL3 and SLC7A11plasmid, the protein expression was checked by western blot analysis.
4. No m6A peak was enriched in SLC7A11 from m6A RIP-seq data.
5. The predicted m^6^A peaks in SLC7A11 mRNA from the m^6^A sites predictor SRAMP.
6. After treatment with Act-D for the indicated times, the mature mRNA levels of SLC7A11 were checked in sh-NC and sh-METTL3 MDA-MB-231cells by qRT-PCR.
7. sh-NC or sh-METTL3 MDA-MB-231 cells were treated with 10 μg/ml CHX for the indicated time periods, and then the protein expression of SLC7A11 was detected by western blot (*left*) and quantitatively analyzed (*right*).
8. Analysis of SLC7A11 mRNA in non-ribosome portion (<40S), 40S, 60S, 80S, and polysome for the METTL3 KD cells compared to control cells.

Data are presented as mean ± SD from three independent experiments. **p*<0.05, ***p*<0.01, ns, no significant, by Student’s *t* test between two groups and by one-way ANOVA followed by Bonferroni test for multiple comparison.

**Related to Figure 2.**

**
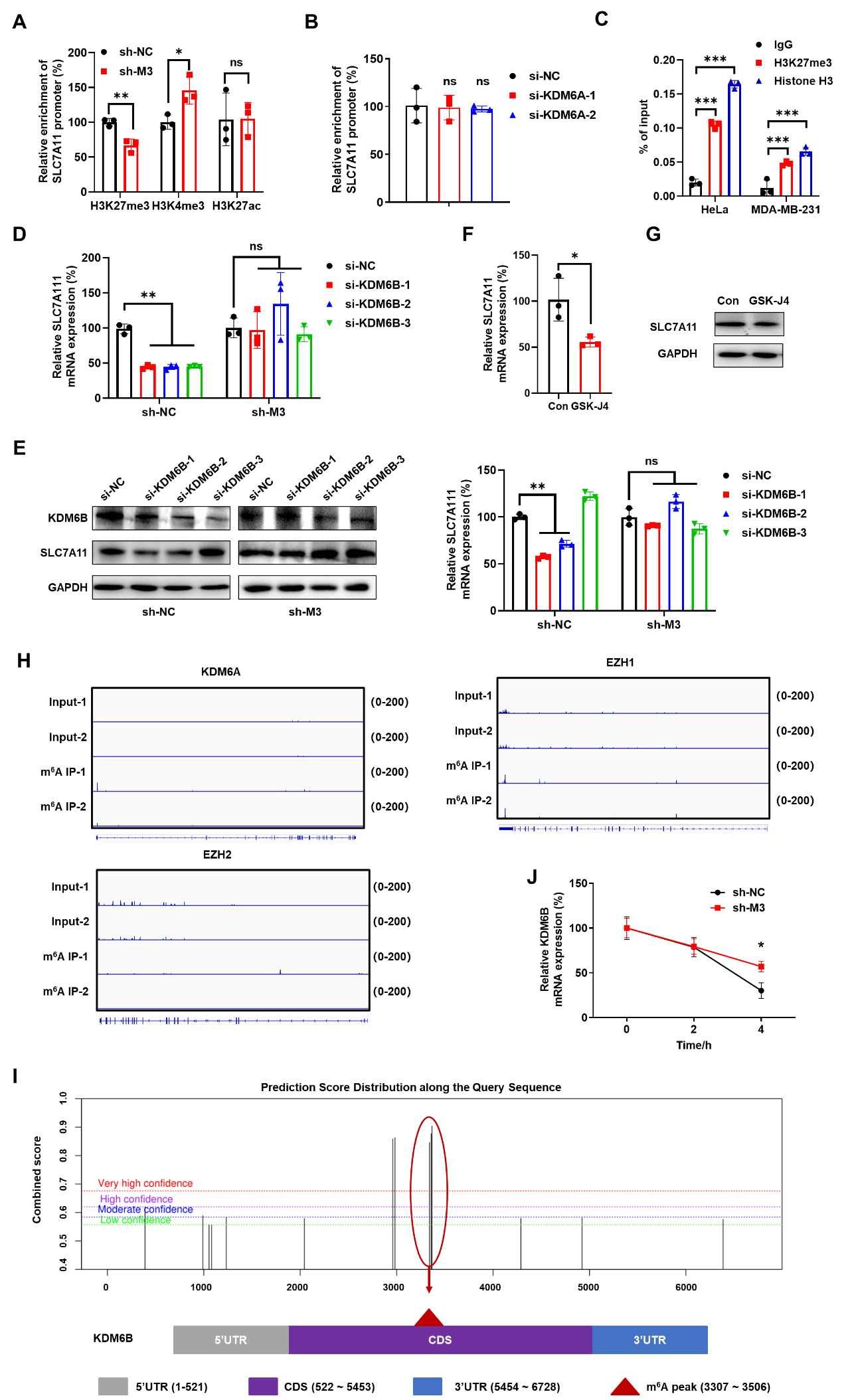
Figure S3.** **METTL3 induced the H3K27 trimethylation of SLC7A11 promoter via suppression of KDM6B.**

1. ChIP-qPCR assay to examine H3K27me3 binding to the SLC7A11 promoter in MDA-MB-231 cells.
2. ChIP-qPCR analysis was performed to examine the binding of H3K27me3 at the promoter region of SLC7A11 after transfecting with siRNAs of KDM6A for 24 h in HeLa cells.
3. ChIP-qPCR assay of the SLC7A11 promoter in HeLa and MDA-MB-231cells. Non-specific rabbit IgG (negative control), H3K27me3 antibody and Histone H3 antibody (positive control) were used in the immunoprecipitation.
4. SLC7A11 mRNA was detected after transfecting with siRNAs of KDM6B for 24 h in MDA-MB-231 cells by qRT-PCR.
5. SLC7A11 and KDM6B protein was detected after transfecting with siRNAs of KDM6B for 48 h in MDA-MB-231 cells by western blot (*left*) and quantitatively analyzed (*right*).
6. After treating HeLa cells with the KDM6B inhibitor GSK-J4(1 μM), SLC7A11 mRNA were assessed by qRT-PCR.
7. After treating HeLa cells with the KDM6B inhibitor GSK-J4(1 μM), SLC7A11 protein were assessed by western blot analysis.
8. m^6^A sequencing data of KDM6A, EZH1, and EZH2.
9. The predicted m^6^A peaks in KDM6B mRNA from the m^6^A sites predictor SRAMP.
10. After treatment with Act-D for the indicated times, the mature mRNA of KDM6B were checked in sh-NC and sh-METTL3 MDA-MB-231 cells.

Data are presented as mean ± SD from three independent experiments. **p*<0.05, ***p*<0.01, ns, no significant, by Student’s *t* test between two groups and by one-way ANOVA followed by Bonferroni test for multiple comparison.

**Related to Figure 3.**


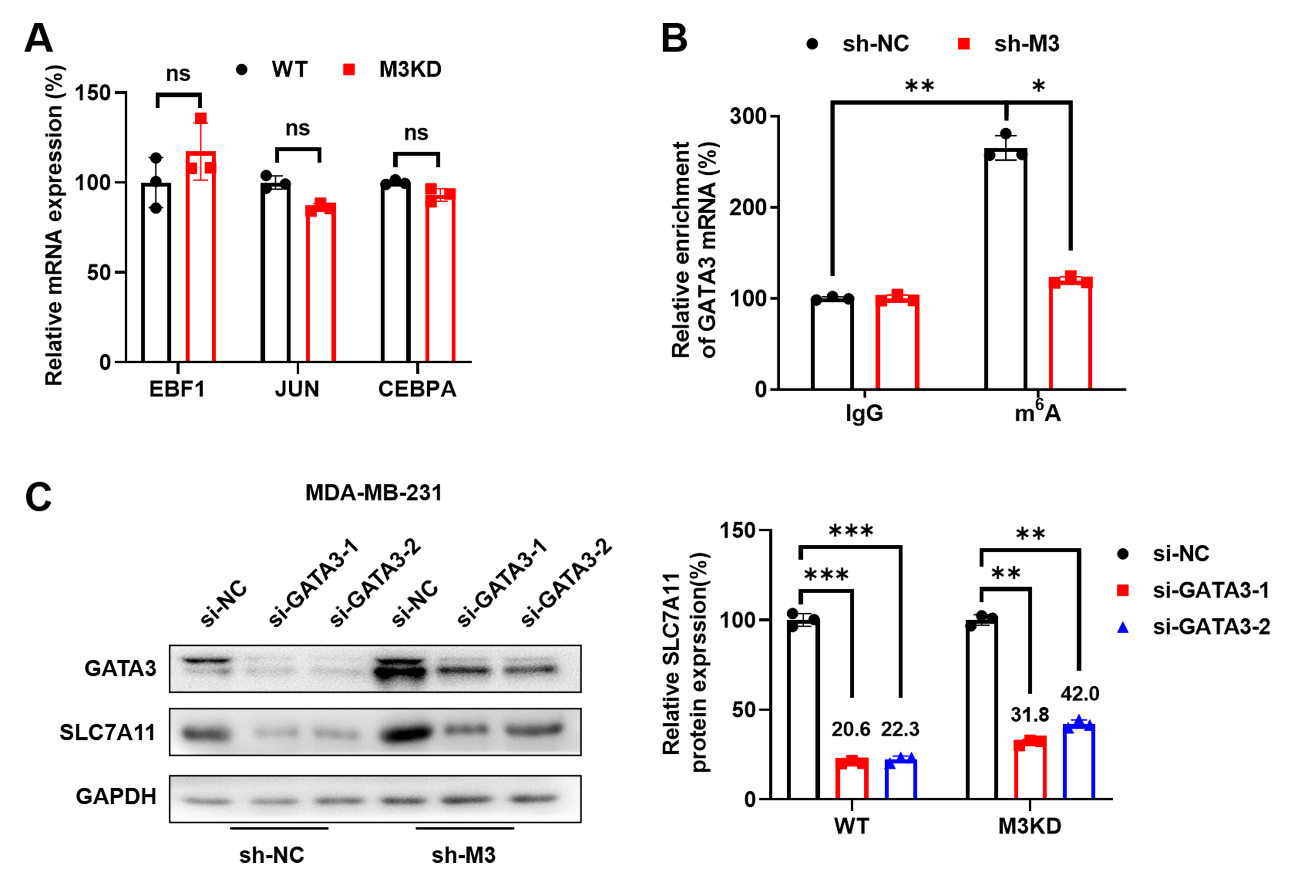


**Figure S4.** **GATA3 was involved in METTL3-regulated transcription of SLC7A11.**

1. The expression of EBF1, JUN and CEBPA mRNA was checked by qRT-PCR in HeLa cells.
2. m^6^A RIP-qPCR analysis of GATA3 mRNA in MDA-MB-231 sh-NC and sh-METTL3 cells.
3. MDA-MB-231 cells were transfected with siRNAs of GATA3 for 48 h, and GATA3 and SLC7A11 protein expression were checked by western blot analysis (*left*) and quantitatively analyzed (*right*).

Data are presented as mean ± SD from three independent experiments. *p<0.05, ***p*<0.01, ****p*<0.001, ns, no significant, by Student’s *t* test between two groups and by one-way ANOVA followed by Bonferroni test for multiple comparison.

**Related to Figure 4.**


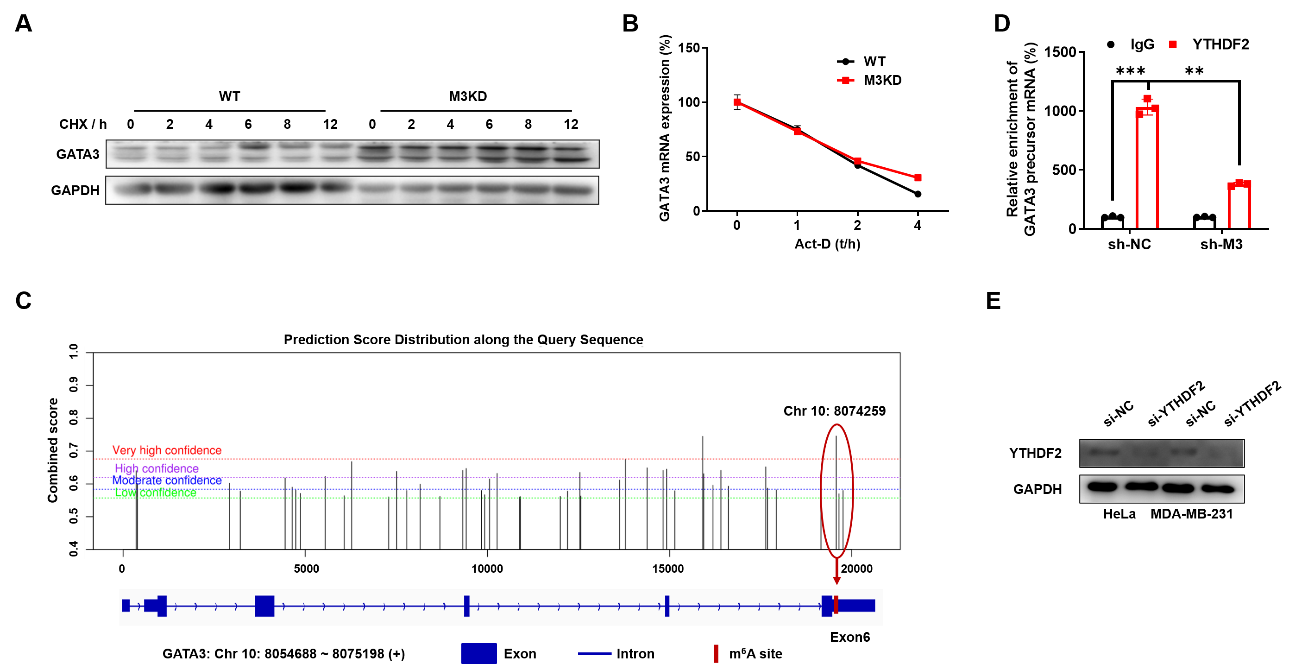


**Figure S5.** **METTL3 decreased precursor mRNA stability of GATA3 via m^6^A dependent recruitment of Dis3L2.**

1. WT or METTL3 KD HeLa cells were treated with 10 μg/ml CHX for the indicated time periods, and then the protein expression of GATA3 was detected by western blot analysis.
2. After treatment with Act-D for the indicated times, the mature mRNA levels of GATA3 were checked in WT and METTL3 KD HeLa cells by qRT-PCR.
3. The predicted m^6^A peaks in GATA3 mRNA from the m^6^A sites predictor SRAMP.
4. YTHDF2 RIP-qPCR analysis of GAT3 precursor mRNA in MDA-MB-231 cells.
5. HeLa and MDA-MB-231 cells were transfected with si-NC or si-YTHDF2, and YTHDF2 protein expression was checked by western blot analysis.

Data are presented as mean ± SD from three independent experiments. ***p*<0.01, ****p*<0.001, ns, no significant, by Student’s *t* test between two groups and by one-way ANOVA followed by Bonferroni test for multiple comparison.

**Related to Figure 5.**


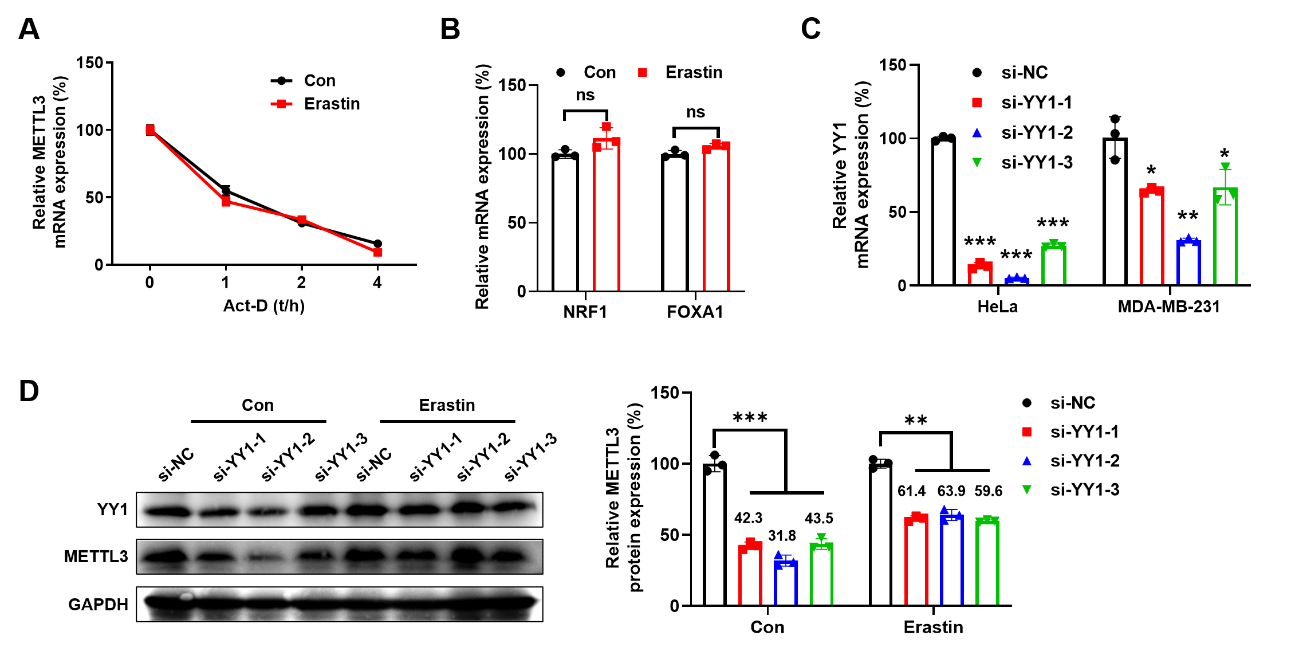


**Figure** S**6. YY1 was responsible for the erastin induced-upregulation of METTL3.**

1. HeLa cells were treated with or without erastin (2μM) for 24 h, and then treated with Act-D for the indicated times. METTL3 mRNA levels were checked by qRT-PCR.
2. HeLa cells were treated with or without erastin (2μM) for 24 h, and then treated with Act-D for the indicated times. NRF1 and FOXA1 mRNA levels were checked by qRT-PCR.
3. YY1 knockdown was achieved using specific siRNA in HeLa(*left*) and MDA-MB-231(*right*) cells, and the mRNA expression of YY1 was subsequently assessed.
4. The expression of YY1 protein was checked by western blot analysis (*left*) and quantitatively analyzed (*right*) in MDA-MB-231 after transfecting with erastin and siRNAs of YY1 for 48 h.

Data are presented as mean ± SD from three independent experiments. **p*<0.05, ***p*<0.01, ****p*<0.001, ns, no significant, by Student’s *t* test between two groups and by one-way ANOVA followed by Bonferroni test for multiple comparison.

**Related to Figure 6.**


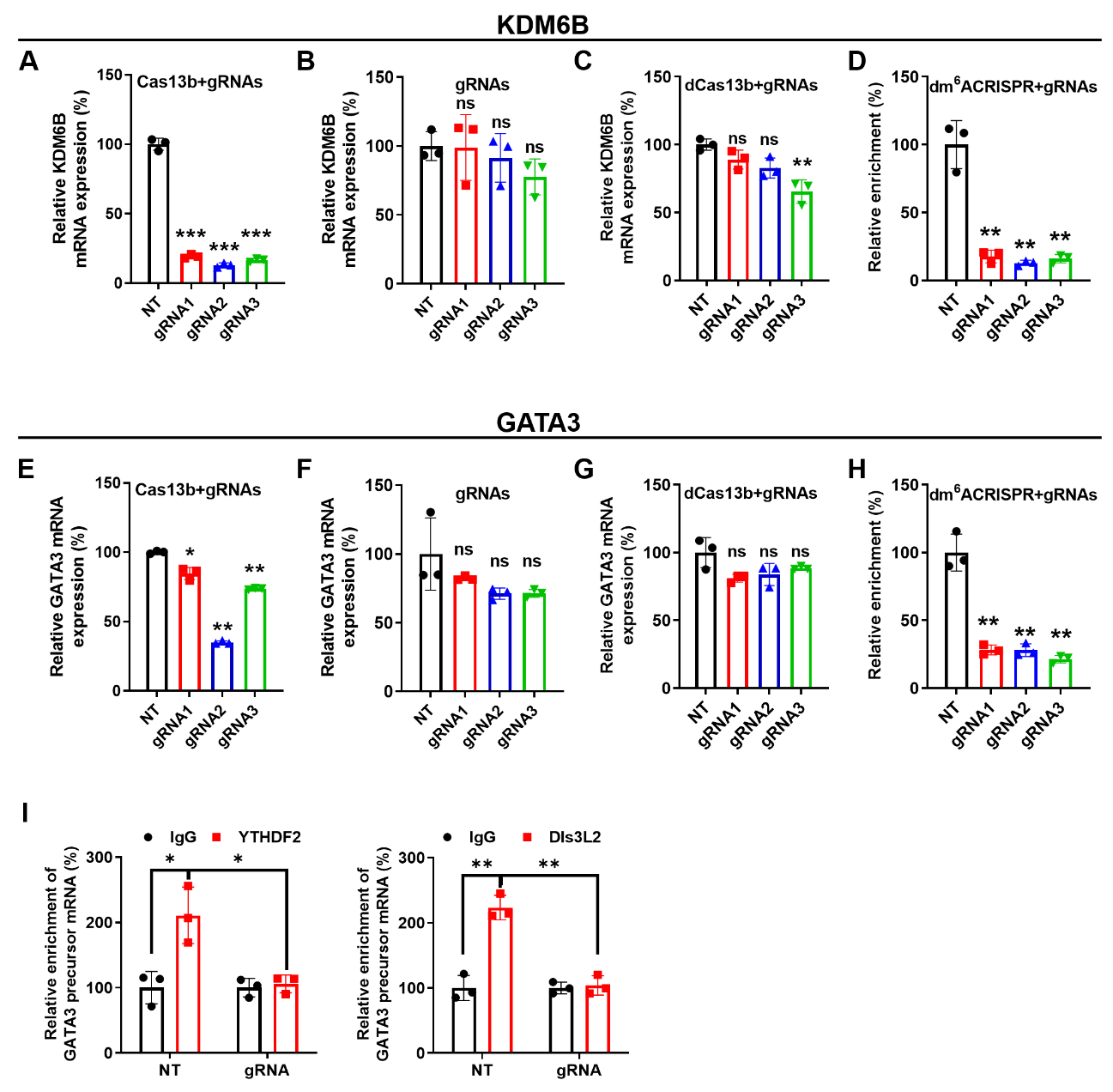


**Figure S7.** **Targeting m^6^A/METTL3 repressed SLC7A11 expression**

1. The mRNA expression of KDM6B in HeLa cells transfected with Cas13b combined with gRNA control or gRNA1/2/3, respectively, for 24 h.
2. The mRNA expression of KDM6B in HeLa cells transfected with gRNA1/2/3, respectively, alone for 24 h.
3. The mRNA expression of KDM6B in HeLa cells transfected with gRNA combined with dCas13b, respectively, for 24 h.
4. m^6^A RIP-qPCR analysis of KDM6B mRNA in HeLa cells transfected with gRNAs and dCas13b-ALKBH5.
5. The mRNA expression of GATA3 in HeLa cells transfected with Cas13b combined with gRNA control or gRNA1/2/3, respectively, for 24 h.
6. The mRNA expression of GATA3 in HeLa cells transfected with gRNA1/2/3, respectively, alone for 24 h.
7. The mRNA expression of GATA3 in HeLa cells transfected with gRNA combined with dCas13b, respectively, for 24 h.
8. m^6^A RIP-qPCR analysis of GATA3 mRNA in HeLa cells transfected with gRNAs and dCas13b-ALKBH5.
9. RIP-qPCR analysis of GATA3 mRNA in HeLa cells transfected with dCas13b-ALKBH5 combined with gRNA for GATA3 mRNA for 24 h by use of antibodies against YTHDF2 and Dis3L2, respectively.

Data are presented as mean ± SD from three independent experiments. *p<0.05, **p<0.01, ***p<0.001, ns, no significant, by Student’s t test between two groups and by one-way ANOVA followed by Bonferroni test for multiple comparison.

**Related to Figure 7.**


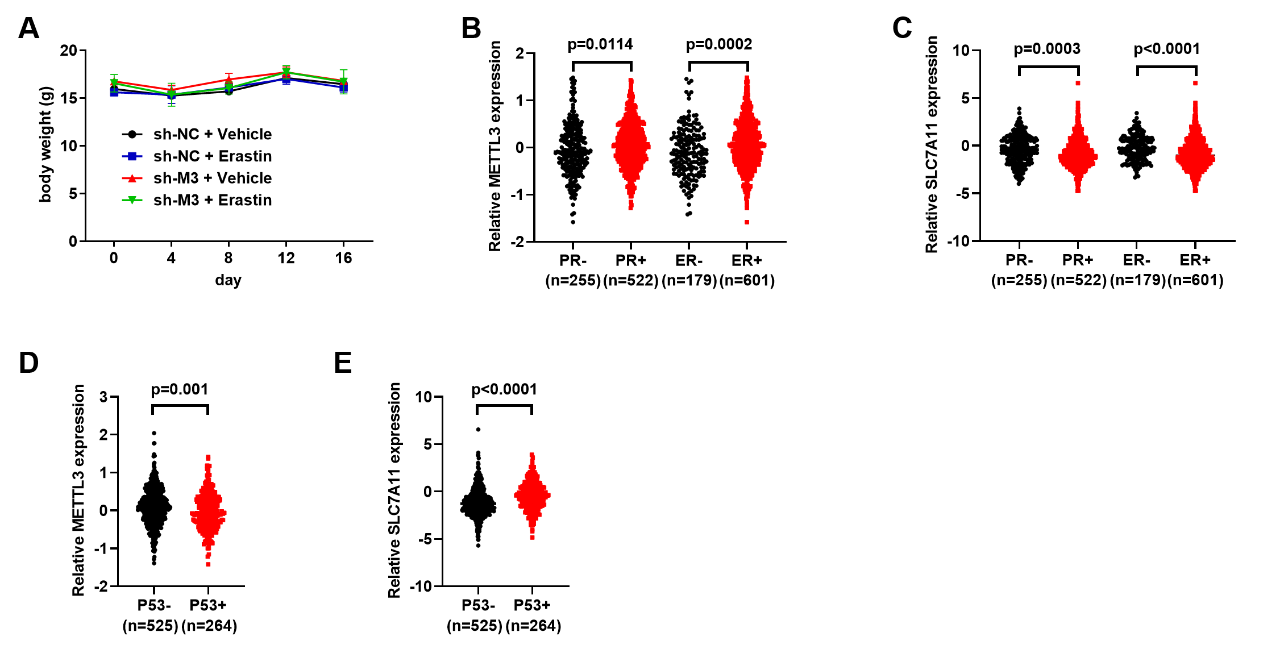


**Figure S8. METTL3/SLC7A11 axis was involved in m^6^A regulated cancer progression**

1. Body weight of xenografts implanted with sh-NC and sh-METTL3 MDA-MB-231 cells and further treated with vehicle or erastin.
2. Expression of METTL3 in Erα and PR negative or positive breast cancer patients from TCGA database.
3. Expression of SCL7A11 in Erα and PR negative or positive breast cancer patients from TCGA database.
4. Expression of METTL3 in TP53-positive BCs as compared with those in TP53-negative breast cancer patients from TCGA database.
5. Expression of SLC7A11 in TP53-positive BCs as compared with those in TP53-negative breast cancer patients from TCGA database.

**Related to Figure 8.**

**Materials and Methods**

1. **LC-MS/MS assay**

mRNA was purified from the total RNA using Poly(A) mRNA Magnetic Isolation Module kit (NEBNext, E7490L). About 200 ng of purified mRNAs were incubated with nuclease P1 (0.5 U, Sigma, N8630) in a 26 μL reaction system containing 20 mM NH4OAc (pH = 5.3) at 42 °C for 2 h, followed by addition of NH4HCO3 (1 M, 3 μL) and alkaline phosphatase (1 μL, 1 U/μL; Sigma, P5931) and incubation at 37 °C for 2 h. Samples were diluted to 80 μl and filtered through a 0.22 μm filter (PALL). All samples (10 μl for each injection) were separated by a C18 column (Agilent) using reverse-phase ultra-performance liquid chromatography and analyzed by an Agilent 6410 QQQ triple-quadrupole LC mass spectrometer using a positive electrospray ionization mode. All nucleosides were quantified by using retention time and ion mass transitions of 268.0 to 136.0 (A) and 282.1 to 150.0 (m^6^A). Quantification was calculated using standard curves from standards running in the same batch. The ratio of m^6^A to A was calculated based on calibration curves.

1. **Colony formation assay**

Cells were plated in a 6-well culture plate at 500 cells per well and grown for 2 weeks in an incubator. 4% paraformaldehyde (Biosharp, BL539A) was added to fix the colonies at room temperature for 15 min. PFA was washed out with water. Colonies were incubated with 1% crystal violet (Beyotime, C0121) for 5 min for staining followed by imaging. The relative number of colonies was quantified by Image J software.

1. **Intracellular GSH and cysteine** **assay**

The relative GSH concentration in cell lysates was assessed using a kit (Beyotime, S0053) according to the manufacturer’s instructions. Oxidized glutathione (GSSG) was reduced to reduced glutathione (GSH) by glutathione reductase. GSH reacted with the chromogenic substrate 5,5'-Dithiobis-(2-nitrobenzoic acid) to produce yellow 5-thio-2-nitrobenzoic acid and GSSG. The GSH level was calculated by measuring absorbance at 412 nm and normalized by protein concentration.

The intracellular cysteine content was detected by a cysteine content assay kit (Solarbio, BC0185). Phosphotungstic acid was reduced to tungsten blue by cysteine, and the tungsten blue exhibited an absorption peak at 600 nm. Cysteine content was calculated by measuring absorbance at 600 nm and normalized by protein concentration.

1. **Polysome profiling**

The fraction of ribosomes was separated by centrifugation in a sucrose gradient. After pretreating with 100 μg/mL cycloheximide, cells were lysed in 600 μL of lysis buffer (140 mM NaCl, 5mM MgCl_2_, 10 mM Tris(pH=8.0), 100 μg/mL cycloheximide, 0.4 U/μL RNase inhibitor, 20 mM DL-Dithiothreitol(DTT), 0.1% cocktail, 10 mM Ribonucleoside Vanadyl Complexes(RVC), 0.5% sodium deoxycholate, 1% Triton-X-100).After centrifugation at 13,000 g for 15 min, the supernatant was separated by 5-50% (w/v) sucrose gradient at 4 °C for 2 h at 170, 000 g (Beckman, rotor SW28). The samples were then fractionated and analyzed by Gradient Station (BioCamp) equipped with an ECONO UV monitor (BioRad) and fraction collector (FC203B, Gilson). The fractions resulting from sucrose gradient were used for RNA extraction and qRT-PCR.

1. **Luciferase reporter assay**

SLC7A11 promoter (−678 to +8) was PCR amplified from genomic DNA derived from HeLa cells using a forward primer (5’-ATG GTA CCT CTG GAG TCC TGG TGA ATT TTG-3’) and a reverse primer (5’-TAA CTC GAG ACA AAC CAG CTC AGC TTC CT-3’) [[1](#_ENREF_1)]. The DNA fragment was cloned into the pGL3-Basic vector using the restriction enzyme cutting sites of Kpn I and Xho I. To generate mutant promoter constructs, the following primers were used: forward, 5’-GAG TAG TAA GAA ATA ATT TT**CC**CT TTT AAT GTT GAG GAA GGC-3’; reverse, GCC TTC CTC AAC ATT AAA AG**GG**AA AAT TAT TTC TTA CTA CTC-3’.

METTL3 promoter reporter was generated in our previous study [[2](#_ENREF_2)]. To generate mutant promoter constructs, the following primers were used: forward, 5’-CCG AGA TCG CGC C**TA** TGC ACT CCA GCC A-3’; reverse, 5’-TGG CTG GAG TGC A**TA** GGC GCG ATC TCG G-3’.

GATA3 3’UTR was PCR amplified from cDNA derived from HeLa cells using a forward primer (5’- TAG CTA GCA GCC CTG CTC GAT GCT CAC-3’) and a reverse primer (5’- GGG TCG ACT AGT TTT AAA ATA TTT TCA CTT TAT TAT TAT GCT TAT AAT ATT ATT CC-3’). The DNA fragment was cloned into the pmirGLO vector using the restriction enzyme cutting sites of Nhe I and Sal I. To generate mutant 3’UTR constructs, the following primers were used: forward, 5’- TGA CTC ATA TCC CCT ATT TA**C** CAG GGT CTC TAG TGC TGT GAA -3’; reverse, 5’-TTC ACA GCA CTA GAG ACC CTG **G**TA AAT AGG GGA TAT GAG TCA -3’.

For the luciferase assay，cells were seeded in 6-well plates in triplicate and incubated for 24 h. Then the pmirGLO-3’UTR plasmid was transfected into cancer cells using Lipofectamine^TM^ 3000 reagent for another 24 h alone, while pGL3 promoter reporter plasmids were co-transfected with pRL-TK Renilla. Firefly and Renilla luciferase signals were detected using the Firefly Luciferase Reporter Gene Assay Kit (Beyotime, RG006) and the Renilla Luciferase Reporter Gene Assay Kit (Beyotime, RG017).

1. **Design of the guide RNAs**

The sequences of all isoforms of target genes were subjected to alignment analysis to identify the common regions, which were considered as targeting candidates for gRNA design. gRNAs targeting GATA3 and KDM6B were designed, and all designed gRNAs were subjected to MEGABLAST (https://blast.ncbi.nlm.nih.gov/Blast.cgi) to avoid mismatching with unexpected targets in the human genome. The sequences of GATA3 gRNAs were: gRNA1, 5′- TGC TGC CTC TGA AAT GCA CAG GCT CTC GCA CAC CAA GAA G-3′; gRNA2, 5′- CAG CAT GTG GCT GGA GTG GCT GAA GGG CGA GAT GTG GCT C-3′; gRNA3, 5′- CTT GTT CAC AAA GCA TGT AGG CCT AGA AAA AGG CTC TCT G-3′. The sequences of KDM6B gRNAs were: gRNA1, 5′- CAG TAC TTT TGT CCC TCC AGC ACG GAG GCC AA-3′; gRNA2, 5′-GCA AGC CCG GCT GAT CTC TTC TAG CAC CTC AG-3′; gRNA3, 5′-CCT GGA TCT CCT CGC TCT GCA GGT CCA GGT TC-3′.

**References**

1. Wang L, Liu Y, Du T, Yang H, Lei L, Guo M, et al. ATF3 promotes erastin-induced ferroptosis by suppressing system Xc(.). Cell Death Differ. 2020;27(2):662-75.

2. Li ZH, Peng YX, Li JX, Chen ZJ, Chen F, Tu J, et al. N-6-methyladenosine regulates glycolysis of cancer cells through PDK4. Nature communications. 2020;11(1).

| **Table S1. 60 Ferroptosis-related genes.** | |
| --- | --- |
| Ferrotosis-related genes | Name |
| ACSL4 | acyl-CoA synthetase long-chain family member 4 |
| AKR1C1 | aldo-keto reductase family 1 member C1 |
| AKR1C2 | aldo-keto reductase family 1 member C2 |
| AKR1C3 | aldo-keto reductase family 1 member C3 |
| ALOX15 | arachidonate 15-lipoxygenase |
| ALOX5 | arachidonate 5-lipoxygenase |
| ALOX12 | arachidonate 12-lipoxygenase |
| ATP5MC3 | ATP synthase membrane subunit c locus 3 |
| CARS | cysteinyl tRNA synthetase |
| CBS | cystathion ine beta synthase |
| CD44 | CD44 molecule |
| CHAC1 | ChaC glutathione- specific gamma-glutamyl cyclotransferase 1 |
| CISD1 | CDGSH iron sulfur domain 1 |
| CS | citrate synthase |
| DPP4 | dipeptidyl-dippeptidase-4 |
| FANCD2 | Fanconi anemia comple mentation group D2 |
| GCLC | glutamate-cysteine ligase catalytic subunit |
| GCLM | glutamate-cysteine ligase modifier subunit |
| GLS2 | glutaminase 2 |
| GPX4 | glutathio ne peroxidase 4 |
| GSS | glutathione synthetase |
| HMGCR | 3-hydroxy-3- methylglutaryl-CoA reductase |
| HSPB1 | heat shock protein beta 1 |
| CRYAB | heat shock protein beta 5 |
| LPCAT3 | lysophosp hatidylcholine acyltransferase 3 |
| MT1G | metallothionein-1G |
| NCOA4 | nuclear receptor coactiva tor 4 |
| PTGS2 | prostagla ndin-endoperoxide synthase 2 |
| RPL8 | ribosomal protein L8 |
| SAT1 | spermidine/spermine N1-acetyltra nsferase 1 |
| SLC7A11 | solute carrier family 7 member 11 |
| FDFT1 | farnesyl-diphosphate farnesyltransferase 1 |
| TFRC | transferrin receptor |
| TP53 | tumor protein 53 |
| EMC2 | ER membrane protein complex subunit 2 |
| AIFM2 | apoptosis inducing factor mitochondria associated 2 |
| PHKG2 | phosphorylase kinase ,g2 |
| HSBP1 | heat-shock 27-k Da protein 1 |
| ACO1 | aconitase 1 |
| FTH1 | ferritin heavy chain 1 |
| STEAP3 | six-transm embrane epithelial antigen of prostate 3 |
| NFS1 | cysteine desulfurase |
| ACSL3 | acyl-CoA synthetase long-chain family member 3 |
| ACACA | Acetyl-CoA carboxylase alpha |
| PEBP1 | phosphatidy lethanolamine-binding protein 1 |
| ZEB1 | zinc finger E-box-binding homeobox 1 |
| SQLE | squalene monooxygenase |
| FADS2 | fatty acid desaturase 2/acyl-CoA 6-desaturase |
| NFE2L2 | nuclear factor, erythroid 2 like 2 |
| KEAP1 | kelch-like ECH- associated protein 1 |
| NQO1 | quinone oxidoreductase-1 |
| NOX1 | NADPH oxidase 1 |
| ABCC1 | ATP binding cassette subfamily C member 1 |
| SLC1A5 | solute carrier family 1 member 5 |
| GOT1 | glutamic-oxaloacetic transaminase 1 |
| G6PD | glucose-6-phosphate dehydrogenase |
| PGD | phosphoglycerate dehydrogenase |
| IREB2 | iron response element-binding protein 2 |
| HMOX1 | heme oxygenase 1 |
| ACSF2 | acyl-CoA synthetase family member 2 |

**Table S2-SLC7A11_TFs**

**Jaspar:**

NFIC ZNF354C CREB1 JUNFOS HOXA5 Gfi1b FOXA1 Foxo1 Foxq1 FOXP2 MEF2A HNF1B MEF2C Nobox Nkx2-5 Nkx3-2 Stat5aStat5b Lhx3 En1 Sox5 Mecom GATA2 Gata1 GATA3 Gata4 Hltf TCF7L2 MAFF MAFK Bcl6 BRCA1 Spi1 Erg SPIB EHF Crx Nr2e3 Mafb FOXI1 Nkx2-5 (var.2) EGR2 Bhlhe40 Meis1 HIF1AARNT ELF5 NKX3-1 Foxa2 FOXO3 ESR2 Pax2 NFE2L1MafG Sox17 CEBPA CEBPB DUX4 MZF1_1-4 Rxra HNF4A HNF4G Foxd3 POU2F2 FOXC1 SOX10 FOXH1 SRY CDX2 BATFJUN FOSL2 JUNB FOS FOSL1 JUND Pdx1 Prrx2 JUN (var.2) PAX5 ArntAhr Atoh1 MZF1_5-13 JUND (var.2) Gfi1 Hoxc9 Hoxa9 ARID3A TBP FOXL1 FOXD1 Sox6 FEV JUN SRF Nr1h3Rxra Stat6 STAT3 Stat4 RUNX1 FOXF2 FOXP1 Sox3 NFIL3 HLF STAT2STAT1 Myb SOX9 Sox2 TFAP2C NR3C1 TEAD1 MAX USF2 NFATC2 IRF1 PRDM1 NFKB1 FLI1 Ets1 GABPA ELK4 ELF1 NFE2MAF ELK1 Myod1 TAL1TCF3 Myog Tcf12 Tcf3 EBF1 STAT1 THAP1 Esrrb NR4A2 Hand1Tcfe2a NFYB AR Klf1 RORA_2 USF1 SREBF1 ZEB1 SP1 Bach1Mafk Nfe2l2 Tcfcp2l1 HSF1 Myc Arnt Mycn RFX5 Ddit3Cebpa Spz1 Pou5f1Sox2 T KLF5 SREBF2 SMAD2SMAD3SMAD4 TP63 NRF1 EGR1 RORA_1.

**PROMO:**

ENKTF-1 GATA2 GATA1 XBP1 GTF2I NR3C1 CEBPB PGR YY1 TBP MEF2A FOXA1 NR3C1 ETS2 IRF1 NFATC2 CEBPA POU2F2 NFATC1 STAT4 ETS1 LEF1 GTF2B NF1 FOXP3 MYB HNF1B HNF1B NR3C1 AR NFYA ELK1 HNF1A TBP NFIA GATA3 POU2F1 SRY TCF4 JUN STAT5A ESR1 NFATC2 STAT1 TCF4 ETV4 THRB TFAP2A EBF1 HOXD9 HOXD10 JUN/FOS VDR RXRA FOS ATF3 PAX5 TP53 NFKB1 RELA HNF4A RXRA RARB NR2F1 USF2 RBPJ.

**ChIP-Atlas:**

EN1 EP300 CEBPG FOXM1 JUNB FOSL1 ESR1 JUN MED1 NR3C1 CEBPA RARA FOS EN1 ATF7 NELFE STAT3 MAU2 PGR GRHL2 TLE3 YAP1 WWTR1 FOXA1 CEBPB MYC BRD4 XBP1 GATA3 PKNOX1 KMT2C SMARCC1 WDR77 HDAC2 KLF9 SMARCA4 TCF7 STAG2 KDM6A CREB1 MNT BRD9 BRD2 Epitope tags TP53 SFPQ RUVBL2 E2F4 ELF1 POLR2C SIN3A CDKN1B SMC1A AR MAFB TRIM25 E2F1 CTCF SMAD3 EBF1 CDYL2.

**Table S3-METTL3_TFs**

**JASPAR:**

Pax2 Arnt Erg NFATC2 SPIB Foxo1 Foxd3 Sox3 Sox6 FOXP1 SRY BRCA1 FOXC1 ARID3A NFIC FOXO3 Hand1 FOXL1 JUND JUN NFE2L1 Hltf Gfi1 Pax2 STAT3 Pdx1 FOXP2 FOXA1 FOXI1 FOSL2 HIF1A Mafb Rxra NR4A2 CEBPA CEBPB Nkx2-5 Prrx2 THAP1 JUN Myb SP1 ZNF354C SREBF1 SREBF2 SOX10 Arnt::Ahr JUN::FOS MZF1_5-13 MEF2C MEF2A Tcf3 ZEB1 EBF1 EGR1 Ddit3::Cebpa Nkx2-5 (var.2) HIF1A::ARNT ELF5 MZF1_1-4 MZF1 KLF5 Stat6 Nkx3-2 HOXA5 Klf4 YY1 NRF1 Gfi1b RUNX1 ELK1 TBP CDX2 Stat4 STAT5 STAT1 ELF1 ELK4 FLI1 FEV GABPA NFE2L1::MafG TFAP2C.

**ChipBase:**

ATF2 BCLAF1 CDX2 CREB1 E2F1 E2F6 EGR1 EHF ELF1 ELF2 ELK1 ELK3 ELK4 ERG ETS1 ETV1 FOXA1 GABPA GMEB2 KDM5A KDM5B KLF4 KLF5 MAX MBD3 MTA3 MYC MYOD1 NKX2-1 NR2F2 NR3C1 NRF1 OVOL2 PAX5 POU5F1 RCOR1 RELA RFX1 SPDEF STAT4 STAT5A TBP THAP1 TP53 USF1 VDR YY1.

**PROMO:**

NO3C1 FOXP3 PAX5 RXRA CEBPB TFIID STAT4 ETS1 MAZ TP53 SP1 THRA CEBPA NFYA NFAT1 ETS2 AP2 FOXE3 RXRB YY1 FOXA1 GTF2I XBPA TBP NRF1 IRF2 GATA1 SRY.

**ChIP-Atlas:**

PARP1 MYC ASXL1 NCAPG E4F1 TEAD1 KMT2C FOXA1 HDAC1 ZHX2 MAX GATA3 JUN BAP1 ZEB1 E2F4 SPDEF ZNF574 ZNF579 EN1 BRD4 KDM6A ACTR5 RBPJ TFAP2C OVOL1 MEN1 WWTR1 ZNF687 GTF2F1 FOS STAT3 MINA GABPA ESR1 ESRRA BANP ZNF444 ELK1 JUNB CBX3 MBD2 CEBPB ZBTB40 ZNF217 SMARCC1 NR2F2 WDR77 HDAC2 EGR1 LIN9 MED1 CTCF ZNF143 HCFC1 NELFE ELF1 SRF BRD2 BRCA1 CREB1 Epitope tags POU2F1 STAT5A E2F1 PML MNT BRD9 XRCC6 ZFX NRF1 AR TP53 SFPQ TCF7 SIN3A YY1 RUVBL2 TCF7L2 POLR2C KDM5B SMC1A NELFA MAFB TRIM25 SMAD3 RAD21 CDYL2.

**Table S4-Targets of interfering sequences**

| **Name** | **Sequence (5’ → 3’)** |
| --- | --- |
| si / sh -METTL3 | GCTGCACTTCAGACGAATT |
| si-GATA3-1 | TCTGCTTCATGGATCCCTA |
| si-GATA3-2 | ACGAGAAAGAGTGCCTCAA |
| si-GATA3-3 | GAAGGCATCCAGACCAGAA |
| si-KDM6A-1 | GAACAGCTCCGCGCAAATA |
| si-KDM6A-2 | GAGAGTAATTCACGAAAGA |
| si-KDM6B-1 | GATGGACTCCAGCGTTTCA |
| si-KDM6B -2 | GGAAGTTTCGAGAGTCCTA |
| si-KDM6B -3 | GTGACAAGGAGACCTTTAT |
| si-YY1-1 | CGACGACTACATTGAACAA |
| si-YY1-2 | CCTGAAATCTCACATCTTA |
| si-YY1-3 | GATGGTTGTAATAAGAAGT |

**Table S5-List of primary antibodies**

| **Name** | **Soure** | **Cat＃NO.** |
| --- | --- | --- |
| METTL3 | Abcam | ab195352 |
| FTO | Abcam | ab126605 |
| AKBH5 | Millipore | ABE547 |
| METTL4 | Abclonal | A9294 |
| GAPDH | Bioworld | AP0063 |
| Dis3L2 | Santa Cruz | sc-517218 |
| KDM6A | Santa Cruz | sc-514859 |
| m^6^A | Synaptic | 202003 |
| SLC7A11 | Proteintech | 26864-1-AP |
| GATA3 | Proteintech | 10417-1-AP |
| YTHDF1 | Proteintech | 17479-1-AP |
| YTHDF2 | Proteintech | 24744-1-AP |
| IGF2BP3 | Proteintech | 14642-1-AP |
| KDM6B | Proteintech | 55354-1-AP |
| YY1 | Proteintech | 66281-1-Ig |
| H3K27me3 | Affinity | DF6941 |
| H3K4me3 | Affinity | DF6935 |
| H3K27ac | Active Motif | 39034 |
| METTL14 | Cell Signaling Technology | 48699 |
| Histone H3 | MedChemExpress | HY-P80166 |

**Table S6-List of primers**

| **Name** | Sequence (5’ → 3’) | **Applications** |
| --- | --- | --- |
| SLC7A11-F | ATGCAGTGGCAGTGACCTT | **qRT-PCR** |
| SLC7A11-R | GGCAACAAAGATCGGAACTG |  |
| ALOX5-F | TGGAATGACTTCGCCGACTTTGAG |  |
| ALOX5-R | GCAGCCATTCAGGAACTGGTAGC |  |
| GCLM-F | GGGCACAGGTAAAACCAAATAG |  |
| GCLM-R | TTTTCACAATGACCGAATACCG |  |
| GAPDH-F | GCACCGTCAAGGCTGAGAAC |  |
| GAPDH-R | TGGTGAAGACGCCAGTGGA |  |
| SLC7A11 precursor-F | GTTTGGGGAAGATGGGAGGG |  |
| SLC7A11 precursor-R | ACCCAACATTCATGCCCGAT |  |
| Firefly-Luc-F | ATCATCCCCGACACCGCTATCC |  |
| Firefly-Luc-R | CCCGAAAGCCGCAGATCAAGTAG |  |
| Renilla-Luc-F | TCAAGGAGAAGGGCGAGGTTAGAC |  |
| Renilla-Luc-R | CCGAAGGTAGGCGTTGTAGTTGC |  |
| KDM6B-F | GCCGGAGTGCCACTGA |  |
| KDM6B-R | ACCCACACTGTCCTTACAGG |  |
| GATA3  5’ UTR-F | CTCTGCTCTTCGCTACCCAG |  |
| GATA3  5’ UTR-R | GGTTGTAAAAAGGGGCGACG |  |
| GATA3  CDS-F | TCACAAAATGAACGGACAGAAC |  |
| GATA3  CDS-R | TTGTGAAGCTTGTAGTAGAGCC |  |
| GATA3  3’ UTR-F | ACCACTGAATCTGGACCCCA |  |
| GATA3  3’ UTR-R | TCTTGGCATCCTTCATGCCTT |  |
| GATA3  precursor-F | CCATGCTGACCATTCTGGGT |  |
| GATA3  precursor-R | AAAACTGTCCCAAGCCAGCT |  |
| YY1-F | CCCACGGTCCCAGAGTCCA |  |
| YY1-R | GTGTGCGCAAATTGAAGTCC |  |
| METTL3-F | CTATCTCCTGGCACTCGCAAGA |  |
| METTL3-R | GCTTGAACCGTGCAACCACATC |  |
| METTL3  precursor-F | CGGAGTTGGTCTAAGAGCCAATT |  |
| METTL3  precursor-R | CAATGGATTGTTCCTTGGCTGTTGTA |  |
| NRF1-F | TGCCGTGGCTGATGGAGAGG |  |
| NRF1-R | GATGCTTGCGTCGTCTGGATGG |  |
| FOXA1-F | GTTCTCCATCAACAACCTCATG |  |
| FOXA1-R | TATTGCAGTGCCTGTTCGTAT |  |
| EBF1-F | GAGTGGAAGCAGCATGAAGGAAG |  |
| EBF1-R | CAGGACGAAGTGGAAGAAGTTGG |  |
| JUN-F | CCAAGAACTCGGACCTCCTCAC |  |
| JUN-F | GCCCTCCTGCTCATCTGTCAC |  |
| CEBPA-F | GACAAGAACAGCAACGAGTAC |  |
| CEBPB-R | TCATTGTCACTGGTCAGCTC |  |
| SLC7A11  Promoter-F | CTCTGGGAAGGTCTGTTCCGAAT | **ChIP-qPCR** |
| SLC7A11  Promoter-R | AGCTCAGCTTCCTCATGGGC |  |
| METTL3 promoter Primer 1 -F | CACCACCTTCAGTGGAAAAGGTATG |  |
| METTL3 promoter Primer 1 -R | CCAGTCTCGAACTCCTCACCTC |  |
| METTL3 promoter Primer 2 -F | CACTTGAACCCGGGAGGT |  |
| METTL3 promoter Primer 2 -R | CGGAGAGGAGTCCTGAGAGT |  |
| METTL3 promoter Primer 3 -F | ACTCTCAGGACTCCTCTCCG |  |
| METTL3 promoter Primer 3- R | GACCCCTGGAGCTGAGCAAG |  |
| F | ATGCAGCGACTCAGCCTCTG | **Select-qPCR** |
| R | TAGCCAGTACCGTAGTGCGTG |  |
